# Retinal neurons establish mosaic patterning by excluding homotypic somata from their dendritic territories

**DOI:** 10.1016/j.celrep.2024.114615

**Published:** 2024-08-11

**Authors:** Christopher Kozlowski, Sarah E. Hadyniak, Jeremy N. Kay

**Affiliations:** 1 Departments of Neurobiology, Ophthalmology, and Cell Biology, Duke University School of Medicine, Durham, NC 27710, USA; 2 Lead contact

## Abstract

In vertebrate retina, individual neurons of the same type are distributed regularly across the tissue in a pattern known as a mosaic. Establishment of mosaics during development requires cell-cell repulsion among homotypic neurons, but the mechanisms underlying this repulsion remain unknown. Here, we show that two mouse retinal cell types, OFF and ON starburst amacrine cells, establish mosaic spacing by using their dendritic arbors to repel neighboring homotypic somata. Using transgenic tools and single-cell labeling, we identify a developmental period when starburst somata are contacted by neighboring starburst dendrites; these serve to exclude somata from settling within the neighbor’s dendritic territory. Dendrite-soma exclusion is mediated by MEGF10, a cell-surface molecule required for starburst mosaic patterning. Our results implicate dendrite-soma exclusion as a key mechanism underlying starburst mosaic spacing and raise the possibility that this could be a general mechanism for mosaic patterning across many cell types and species.

## In brief

Evenly spaced “mosaics” of retinal neurons were discovered a century ago, but how mosaics form remains unknown. Here, Kozlowski et al. show that developing mouse starburst amacrine cells use MEGF10, a repulsive cell-surface molecule, to exclude neighboring somata from their dendritic territories. This repulsion establishes uniform spacing within the mosaic.

## Graphical abstract

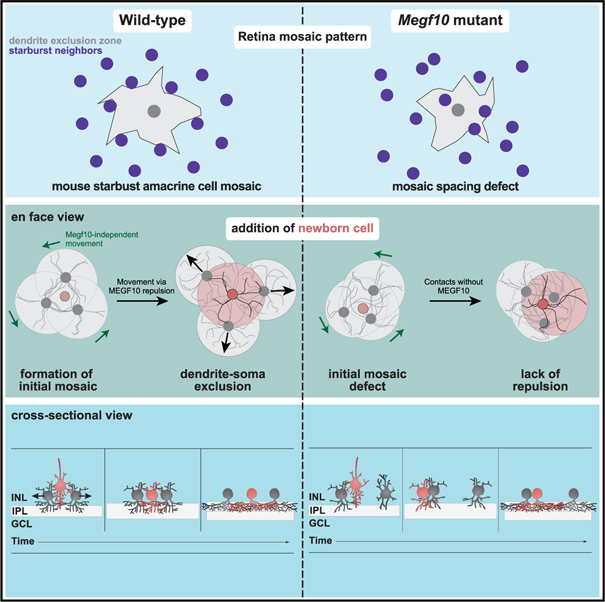

## INTRODUCTION

The vertebrate retina contains >120 cell types, each specialized for particular visual functions.^1^ Many of these exhibit mosaic patterning, whereby individual neurons of a given type are distributed into regularly spaced soma arrays.^2^ This patterning system is crucial for visual processing: it ensures uniform and complete distribution of neural elements across the retina so that the same computations may be performed throughout the visual field. Accordingly, disruption of mosaic patterning impairs retinal circuit function and visually guided behavior.^3,4^ Because mosaics are so fundamental to retinal anatomy and functional output, it is critical to understand how they arise during development.

The most important developmental phenomenon driving mosaic patterning is local cell-cell repulsion between neurons of the same type.^5–7^ Such repulsion produces one of the defining features of mature mosaics: the “exclusion zone”—a region surrounding each cell where another homotypic neuron is rarely found.^8,9^ Mature mosaics may also exhibit regularity over larger spatial scales; however, modeling studies suggest that local repulsion suffices to impose global order upon the entire neuronal array.^10–13^ Thus, the key to understanding mosaic patterning is to define the mechanisms by which homotypic neurons repel each other to produce exclusion zones. At present, these mechanisms remain unclear.

**Figure 1.**
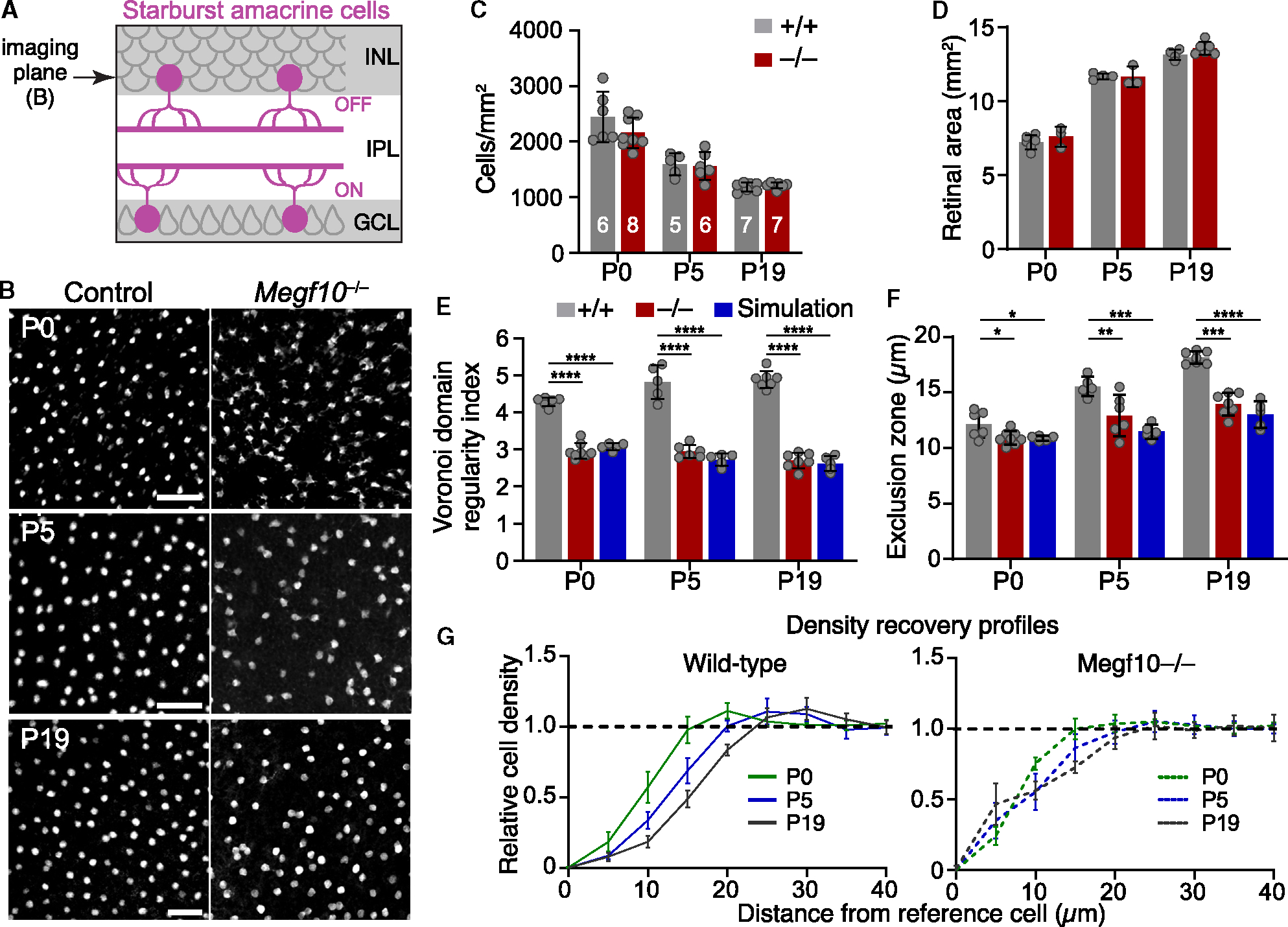
Early establishment of OFF starburst mosaic spacing requires MEGF10. (A) Schematic of mouse retina, cross-sectional view. Starbursts with dendrites in inner plexiform layer (IPL). INL, inner nuclear layer (OFF starbursts); GCL, ganglion cell layer (ON starbursts). Arrow, *en face* imaging plane (e.g., in B) for OFF starburst mosaic patterning analysis. (B) OFF starburst cell arrays in *Megf10*^−/−^ mutants and wild-type littermate controls (*Megf10*^*+/+*^). Starbursts were labeled using the following: P0, anti-Sox2 (wild-type) or anti-β-galactosidase (mutant, *Megf10*^*lacZ*^); P5, anti-Sox2; and P19, anti-ChAT. Also see Figure S1A for comparison to random arrays. (C and D) OFF starburst cell density (C) and retinal area (D). Density data (C) are averaged from 3 mid-peripheral images per animal. Numbers of animals are indicated on graph. (E) Regularity of OFF starburst array, assessed by VDRI (see Figure S1), in real images (gray and red) and in simulated random arrays matched in cell size and density to real data (blue). Statistics: 1-way ANOVA (main effects of genotype at each age, *p* <1 × 10^−7^) with Tukey’s post-hoc test, ****p <1 × 10^−6^. *p* values for mutant vs. simulated arrays: P0, *p* = 0.43; P5, *p* = 0.44; and P19, *p* = 0.82. (F and G) Exclusion zone sizes of OFF starbursts (F), measured using density recovery profiles (DRP; see Figure S1). Wild-type DRP curves (G) show starburst density is lower at short spatial scales than global density (dashed line) demonstrating short-range repulsion. Exclusion zones become larger with time (F, see results for statistics; G, rightward shift of DRP curve). *Megf10* exclusion zone sizes were indistinguishable from random arrays at all ages (F). Statistics (F): 1-way ANOVAs were used to test for genotype effects at each age (main effect of genotype at P0, *p* = 0.019; at P5, *p* =9 × 10^−4^; and at P19, *p* >1 × 10^−7^) with Tukey’s post-hoc test, *p < 0.05; **p < 0.01; ***p < 0.001; ****p <1 × 10^−6^. *p* values for mutant vs. simulated arrays are as follows: P0, *p* = 0.99; P5, *p* = 0.19; and P19, *p* = 0.23. (C–G) Number of mice are indicated in (C). Simulations, *n* = 5. Error bars, SD. Scale bars: 50 μm. Also see Figure S1.

Here, to investigate mechanisms underlying homotypic repulsion and exclusion zone formation, we used the cholinergic “starburst” amacrine cells of mouse retina as our model (Figure 1A). Starburst cells are advantageous for this purpose not only because they have a long history as a model for studying mosaics,^2,14^ but also because they are among the only cell types for which the molecular mechanism driving exclusion zone formation is known.^15^ Two types of starburst neurons form independent mosaics (Figure 1A): OFF starbursts of the inner nuclear layer (INL) and ON starbursts of the ganglion cell layer (GCL). Patterning of both starburst mosaics depends on the cell-surface protein MEGF10, which is selectively expressed by starburst cells and serves as both receptor and ligand to mediate homotypic recognition and repulsion.^4,15^ In mice lacking *Megf10* gene function, starburst positioning is no longer constrained by the locations of homotypic neighbors; instead, soma positions are random, indicating a complete loss of exclusion zones.^15^ Moreover, MEGF10-mediated repulsion is sufficient to generate ectopic exclusion zones, as MEGF10 misexpression causes starbursts to be repelled away from non-starburst misexpressing cells.^15^ Thus, MEGF10 must control the key cellular events that dictate exclusion zone formation. However, those MEGF10-dependent cellular events remain unknown. Defining these MEGF10-dependent events will provide insight into the cellular mechanisms by which neurons avoid each other during exclusion zone formation.

To learn how MEGF10 mediates starburst cell-cell repulsion, we focused on the developmental period shortly after neurogenesis, when newborn neurons complete their radial migration across the outer neuroblast layer to their final laminar position at the inner plexiform layer (IPL).^14^ Prior research has shown that this is the time when homotypic contacts are first established, likely driven by onset of dendritic outgrowth, which coincides with completion of radial migration.^12,16^ These homotypic contacts induce tangential migrations that establish exclusion zones.^12,17–19^ However, the anatomical nature of the contacts and the mechanisms driving tangential movement once contact has occurred remain unclear. Dendritic tiling, a phenomenon whereby neurons establish nonoverlapping dendritic territories using homotypic dendritic repulsion,^20^ was proposed as a cellular mechanism for establishing exclusion zones.^6,16,21^ While tiling is fairly uncommon in adult retina,^9^ mouse horizontal cell dendrites tile transiently during early development, when mosaic spacing is being established.^21^ Based on this finding, transient tiling is now widely considered to be the most likely cellular mechanism underlying exclusion zone formation. Nevertheless, the transient tiling model has yet to be critically tested alongside other potential forms of homotypic repulsion.

Here, we built a mouse genetic toolkit for visualizing starburst anatomy at the earliest stages of their differentiation, and we used it to identify MEGF10-dependent cellular events that occur when starbursts are establishing their exclusion zones. We demonstrate that, instead of tiling, starburst dendrites transiently contact the somata of their starburst neighbors. These contacts lead to establishment of dendritic territories from which neighboring homotypic somata are excluded. In *Megf10* mutants, starburst dendrites still contact neighboring homotypic somata, but these contacts cannot prevent those cells from residing within their arborization territory. Our findings therefore support a model of mosaic formation whereby dendrite-soma repulsion establishes exclusion zones. This model applies to both OFF and ON starburst populations, and based on previous anatomical observations, it may generalize as a mosaic patterning mechanism for other cell types in mouse and primate retina.^22,23^

## RESULTS

### OFF starburst exclusion zones emerge by the day of birth

To investigate the cellular mechanisms by which starburst amacrine cells become patterned into a mosaic, we began by defining the developmental period when exclusion zones first arise. To this end we evaluated development of the OFF starburst mosaic at three time points. We analyzed both wild-type mice and *Megf10* null mutants, which lack OFF starburst exclusion zones at maturity.^15^ Starburst arrays were imaged *en face* in whole-mount retinas stained for starburst markers Sox2^4,24^ or choline acetyltransferase (ChAT; Figure 1B). Spatial properties of the arrays were quantified using two standard methods (illustrated in Figure S1). Array regularity was measured using the Voronoi domain regularity index (VDRI)^25,26^; and the density recovery profile (DRP) was used to measure exclusion zone size.^27^ The extent of regularity (VDRI) or cell-cell avoidance (DRP) at each time point was determined by comparing real starburst data to measurements made on simulated 2D arrays of randomly distributed cells, matched to the cell size and density of the real data (Figure S1). In these random simulations, the only constraint on cell position was that two cells cannot occupy the same physical location. Therefore, for the random arrays, the exclusion zone size is equal to the starburst soma diameter.^9,15^ By contrast, real starbursts—which are not randomly distributed—show larger exclusion zones and more regular VDRI values than the random simulations^15,28^ (Figures 1E, 1F, and S1).

Developmental changes in OFF starburst mosaic patterning were evaluated across three time points: postnatal day (P)0, P5, and P19. In wild-type mice, exclusion zones and orderly starburst positioning were already present at P0 (Figures 1E and 1F). As the retina continued to grow between P0 and P19 (Figure 1D), exclusion zones became larger (Figures 1F and 1G; one-way ANOVA, main effect of age *p* < 1 × 10^−7^), and mosaic regularity improved (Figure 1E; one-way ANOVA, main effect of age *p* = 0.004). By contrast, in *Megf10* mutants, starburst positioning was indistinguishable from random simulations at all ages, and exclusion zones failed to emerge as the retina expanded (Figures 1E–1G and S1A). Absence of exclusion zones was not due to effects on retinal area or cell density, as mutants were comparable to wild-type controls on both measures (Figures 1C and 1D). These results demonstrate that MEGF10-dependent cell-cell interactions driving starburst exclusion zone formation begin prior to P0 and continue into the early postnatal period.

### *Megf10*^*Cre*^ and *Chat*^*Cre*^ mouse lines reveal starburst anatomy during mosaic patterning

**Figure 2.**
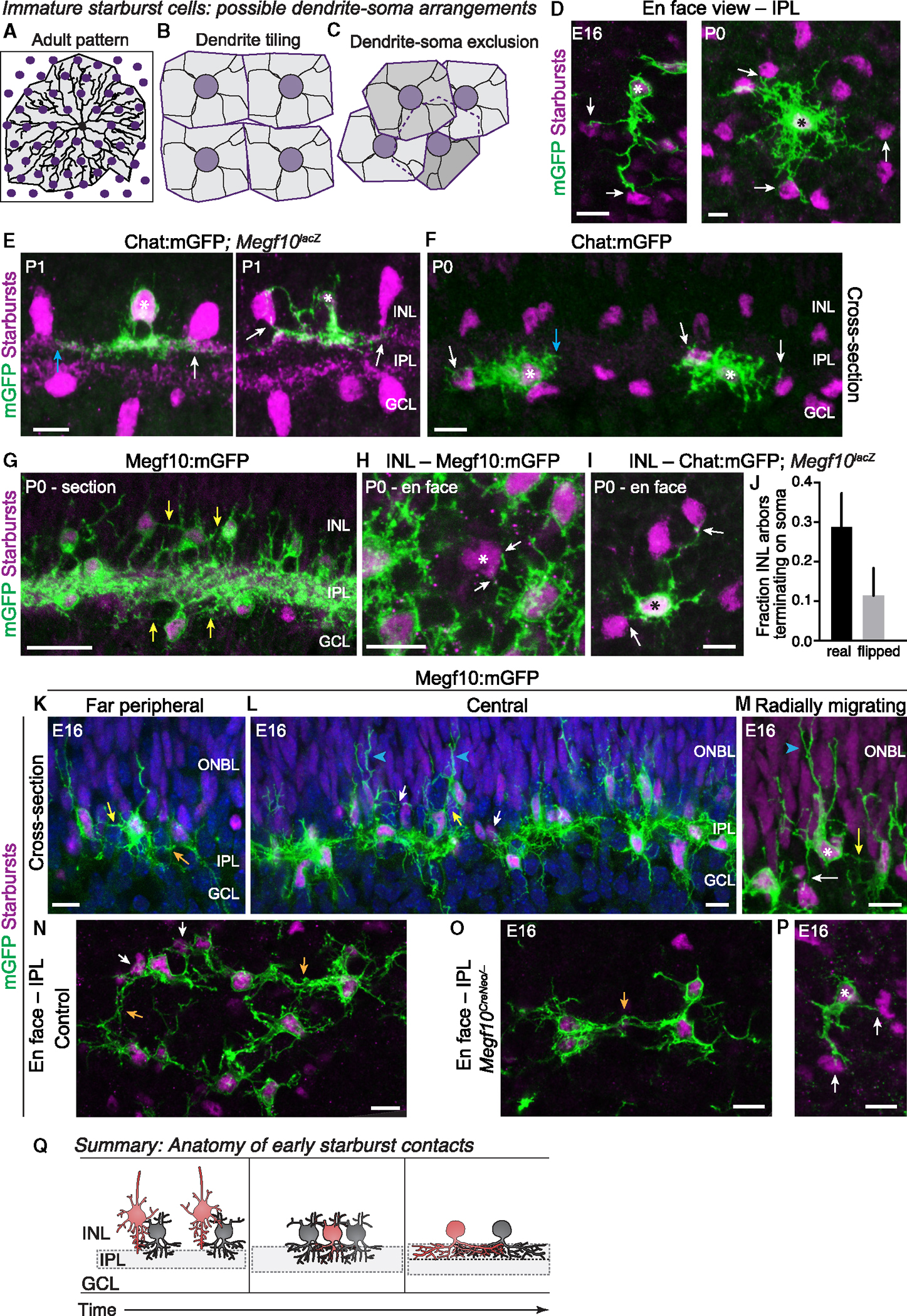
Nascent starburst dendrites contact neighboring starburst somata. (A–C) Model for how starburst dendrites (black) might be positioned relative to neighboring starburst somata (purple). (A) Adult pattern: starburst dendrite territories (gray shading) extend beyond adjacent homotypic neighbors. (B) Transient tiling: homotypic repulsion at dendrite tips establishes nonoverlapping territories to generate exclusion zones. (C) Dendrite-soma exclusion: repulsion between dendrites and somata prevents neighboring cells from residing within dendritic territories. (D) Single mGFP^+^ starburst neurons (asterisks) at E16 (left; Megf10:mGFP) or P0 (right; Chat:mGFP). Sox2 (magenta) labels all starbursts. Note similarity of real images to model (C). (E and F) Dendro-somatic interactions between OFF (E) and ON (F) starburst cells at P0–P1. Asterisks, Chat:mGFP^+^ cells; all starbursts, anti-βgal in *Megf10*^*lacZ*^ mice (E) or anti-Sox2 (F). GFP^+^ dendrite tips contact somata (white arrows) or are aligned with them (blue arrows). Image in (E) (right panel only) is reproduced from Ray et al.^4^ (G and H) P0 starburst dendritic projections labeled by Megf10:mGFP. (G) Cross-section; (H) *en face* whole-mount view of INL arbors. A rare GFP-negative Sox2^+^ starburst cell (asterisk) is contacted by GFP^+^ dendrites (arrows). (I) INL arbors of an individual Chat:mGFP^+^ starburst, contacting a neighboring Sox2^+^ cell (arrow). (J) Quantification of fraction GFP^+^ arbor tips terminating on GFP-negative starburst somas using Chat:mGFP images similar to (I). Chance contact rate was determined from control images flipped about the X and Y axes (“flipped”). Sample size, 122 arbors from 22 cells. Error bars, 95% CI. (K and L) Megf10:mGFP starburst anatomy at E16 in far periphery (K) or central retina (L). Sox2, starburst nuclei. (K) Note soma-layer contacts (also see D for definitive E16 dendrite-soma contacts) and co-fasciculation of IPL dendrites. (L) Blue arrowheads, later-arriving cells with radial migratory morphology. Processes from migrating cells contact established cells (arrows). (M) Higher-magnification view of E16 GFP^+^ starburst cell with radial morphology (blue arrowhead) making soma contacts (arrows). (N and O) E16 starburst anatomy in control (N, *Megf10*^*CreNeo/+*^) and *Megf10* mutant (O, *Megf10*^*CreNeo/−*^), labeled by Megf10:mGFP. Images are Z-projections acquired at IPL level. Starburst dendrites of both genotypes are unipolar or bipolar in tangential plane, forming dendritic fascicles (orange arrows) that interconnect adjacent starbursts. White arrows, dendrite-soma contact. (P) Individual E16 *Megf10* mutant starburst from far peripheral Megf10:mGFP retina. Note resemblance to E16 control starburst (D, left). (Q) Schematic summarizing anatomy findings: (left) embryonic radially migrating starbursts (red) contact established somata (gray). After migration (center), starbursts elaborate arbors through both IPL and soma layers without tiling, instead contacting neighboring somata. During early postnatal period (right), soma-layer arbors are eliminated, but dendrite-soma alignment remains. White arrows (all panels), contact between GFP^+^ dendrites and GFP^−^ somata, confirmed in z stacks. Yellow arrows, putative dendrite-soma contacts between GFP^+^ dendrites and GFP^+^ somata. Orange arrows, arbor fascicles within IPL. Scale bars: 25 μm (G); 10 μm (all other panels). Also see Figure S2.

We next sought to determine the anatomical nature of the cell-cell interactions underlying starburst exclusion zone formation. Addressing this question required development of tools to reveal anatomy of starburst cell-cell contacts during the perinatal period, when exclusion zones are established (Figure 1). We previously showed that *Chat*^*Cre*^ mice are useful for sparse labeling of starburst neurons at early postnatal stages.^4^ Thus, to study anatomical details of single starburst neurons at P0–P3, we crossed *Chat*^*Cre*^ mice to Cre-dependent reporter mice expressing membrane-targeted green fluorescent protein (denoted Chat:mGFP; Figures 2D–2F and 2I). As a complementary tool, we generated *Megf10*^*Cre*^ mice that label the full starburst population at P0 (Figures 2G and S2B), as well as labeling starbursts at embryonic stages (Figures 2D, 2K–2P, and S2G). Two alleles were generated: a parental *Megf10*^*CreNeo*^ allele, in which retention of a neomycin selection cassette abrogates *Megf10* gene function, and *Megf10*^*Cre*^, in which removal of the FRT-flanked selection cassette restored *Megf10* functionality (Figures S2A and S2D–S2F). Crosses to mGFP reporter mice (Megf10:mGFP labeling) revealed that both lines faithfully recapitulate endogenous *Megf10* expression^4,15^ (Figures S2B–S2E), including onset of expression as embryonic starbursts are completing their radial migration (Figures 2L and 2M). Therefore, Chat:mGFP and Megf10:mGFP mice together provide the necessary tools to visualize starburst anatomy during the perinatal period when exclusion zones are established.

### Starburst dendrites contact homotypic somata during mosaic formation

Using these mouse lines, we first examined whether the anatomy of perinatal starburst neurons is consistent with the prevailing “transient tiling” model of exclusion zone formation (Figures 2A and 2B). This model holds that the key homotypic repulsive interactions occur at dendritic tips (Figure 2B). Using Chat:mGFP single-cell labeling in cross-sections and whole-mounts, we found that starburst anatomy at P0–P1 was inconsistent with tiling. Rather than stopping at their neighbor’s dendritic tips, starburst arbors within the IPL frequently extended all the way to neighboring starburst cell bodies (Figures 2D–2F). Alignment between dendrite tips in the IPL and neighboring somata could be remark ably precise (Figures 2E and 2F). For OFF starbursts, contacts were typically localized to the base of the neighboring starburst soma where it touched the IPL (Figure 2E), whereas ON starburst arbors typically ramified at the border of the IPL and GCL, enabling some branches to enter the GCL and contact starburst somata (Figure 2F). In some cases, OFF starburst dendrites also branched out of the IPL to contact INL cell bodies (Figure 2E, right panel). Together, these observations raised the possibility that the key sites of homotypic repulsion leading to exclusion zone formation are not at dendritic tips but instead involve contact between dendrites and neighboring somata (see schematic, Figure 2C).

In addition to their IPL arbors, P0–P1 starbursts also produce a transient dendritic arborization within their cell body layer—i.e., the INL or GCL—that is eliminated by P3^4^ (Figures 2E–2I and S2). We previously showed that this transient soma-layer arbor network consists almost entirely of homotypic contacts among starburst cells,^4^ suggesting it could be a site of homotypic interactions relevant to exclusion zone formation. We therefore asked whether these soma-layer dendrites tile. If so, dendrite-soma contacts should be negligible (Figure 2B). To the contrary, however, we observed numerous apparent dendrite-soma contacts using both bulk (Megf10:mGFP; Figures 2G and 2H) and single-cell labeling (Chat:mGFP; Figures 2E, 2F, and 2I). To measure how frequently dendrite-soma contacts occur within the INL, we combined Chat:mGFP single-cell labeling with a marker that reveals the complete starburst population (anti-β-galactosidase in an *Megf10*^*lacZ*^ background; Figures 2E–2I). Using z stacks from retinal whole mounts (Figure 2I), each GFP^+^ dendritic tip was evaluated for starburst homotypic contact—both in real images and in control images in which the GFP channel was flipped about both axes, to measure the contact rate expected by chance. Contrary to the tiling model, we found that ~30% of INL dendrites terminate on a starburst cell body (Figure 2J), and that 73% of cells in our dataset had at least one soma-contacting dendrite (*n* = 16/22). Moreover, the observed 30% contact rate is higher than the rate expected by chance (Figure 2J), suggesting that soma-layer dendrites selectively target neighboring somata rather than touching them coincidentally. Together, these results indicate that transient soma-layer arbors of P0–P1 starbursts behave quite similarly to their IPL arbors: In both cases, dendrites do not tile but instead tend to contact somata of homotypic neighbors.

We next tested whether starburst tiling might occur even earlier—during embryonic stages. At embryonic day (E)16, most starbursts were labeled by Megf10:mGFP, although in less mature regions at the retinal periphery, some Sox2^+^ starbursts did not yet express GFP (Figures 2K–2N). This center-to-peripheral gradient in reporter expression allowed us to assess both single-cell starburst morphology as well as interactions among adjacent GFP^+^ starburst neurons. Additionally, center-to-peripheral gradients in developmental timing enabled our E16 analysis to capture a range of starburst maturation stages. In far peripheral retina, the least mature retinal region, GFP^+^ starbursts had a multipolar morphology with minimally branched arbors extending in many directions—including into the IPL but also into the soma layers (Figure 2K). Rather than tiling, these rudimentary arbors typically extended to reach the location of neighboring starburst somata and frequently contacted them (Figures 2D and 2K). In central regions, where E16 retina is more mature, starbursts had begun to ramify nascent dendrites within the IPL forming a discontinuous IPL network, although soma-layer contacts were still prominent (Figures 2L and S2G). Viewed *en face*, the anatomy of these IPL contacts was inconsistent with tiling: rather than forming a space-filling network—the pattern expected under a tiling model (Figure 2B)—E16 IPL projections had a unipolar or bipolar morphology, with extensive co-fasciculation (Figure 2N). Furthermore, we again observed IPL dendrites that contacted starburst cell bodies (Figure 2N, white arrows).

Finally, in central E16 retina, we evaluated how newly generated Megf10:mGFP^+^ starburst cells interacted with more mature cells that arrived earlier. Newly generated cells were identified based on their radial morphology, their lack of IPL innervation, and their greater distance from the IPL—all of which indicated that these cells were still radially migrating (Figures 2L and 2M). Even at this migratory stage, starbursts already possessed neurites that contacted adjacent homotypic somata (Figures 2L and 2M). Altogether, these anatomical observations indicate that starburst dendrites can directly target somata without first moving through a phase of transient tiling (Figure 2Q). Such contacts occur until at least P1, both in soma layers and IPL. Furthermore, even dendrites that no longer touch neighboring somata remain aligned with them (Figure 2Q), suggesting the existence of dendrite-soma exclusion (Figure 2C).

### OFF starburst dendrites exclude neighboring somata from their territories

Based on the observed anatomy during the perinatal period (Figures 2C, 2D, and 2Q), we hypothesized that dendrite-soma contacts establish exclusion zones by transiently restricting starburst somata from entering another cell’s dendritic territory. If this model is correct, we can make the following two predictions: (1) During mosaic formation, starburst arbors should demarcate a zone within which neighboring cells are rarely found; and (2) this dendro-somatic avoidance behavior should be impaired in *Megf10* mutants, which lack starburst exclusion zones (Figure 1F).

**Figure 3.**
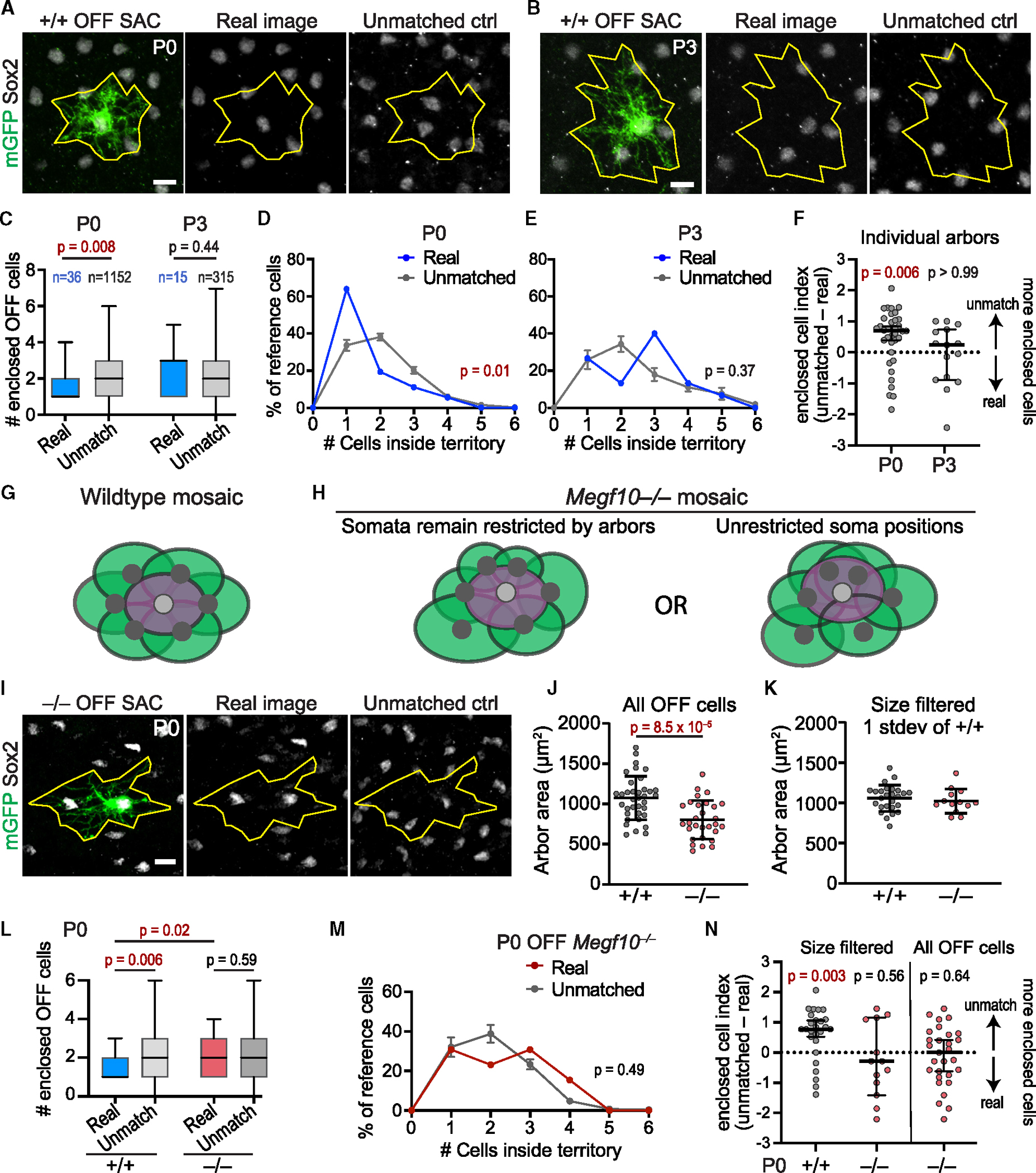
OFF starburst dendritic arbors exclude homotypic somata in a MEGF10-dependent manner. (A and B) Representative wild-type (+/+) Chat:mGFP^+^ OFF starburst amacrine cells (SACs) at P0 (A) or P3 (B). Real images (left and center) show GFP^+^ reference cell dendritic territory (yellow) in relation to neighboring Sox2^+^ starbursts. In unmatched control (“ctrl”) image, dendritic polygon was placed onto OFF starburst array from a different image. (C) Summary of OFF Sox2^+^ cells fully enclosed by real dendritic territory polygons (blue) or unmatched controls (gray). Box, interquartile range; black line, median. Sample sizes are as follows: P0, *n* = 36 real arbors from 7 animals; *n* = 1,152 control images (32 unmatched images per real arbor). P3, *n* = 15 real arbors from 3 animals; *n* = 315 control images (21 unmatched images per arbor). Statistics: two-tailed Mann-Whitney test. (D and E) Fraction of reference cells enclosing *n* somata at specified age. Sample sizes are as follows: real arbors as in (C); unmatched P0, *n* = 1,117; unmatched P3, *n* = 297 (zero values excluded). Statistics: chi-squared test. (F) Individual P0 arbors enclose fewer Sox2^+^ cells than their own unmatched controls, as shown by the enclosed cell index. P3 arbors enclosed Sox2^+^ cells at the chance rate (dashed line). Sample sizes are as in (C). Statistics: Wilcoxon one-sample test with theoretical median of 0. (G) Illustration of how dendrite-soma exclusion produces even spacing in wild-type. Shaded circles, dendrite territories. (H) Schematics illustrating two possible explanations for random cell positioning in *Megf10* mutants. Left, dendrite-soma exclusion remains intact, resulting in variable arbor sizes (green). Right, mutant somata are no longer excluded from neighboring arbor territories. (I) Representative Chat:mGFP^+^ reference cell from P0 *Megf10*^−/−^ retina, shown with its real Sox2^+^ starburst array (center) and with an unmatched OFF starburst array (right). (J) Dendritic territories in mutant P0 OFF starbursts (*n* = 31 arbors from 7 *Megf10*^−/−^ animals) are smaller than littermate controls (*n* = 36; see B). Statistics: two-tailed t test. (K) Filtering of arbors >1 standard deviation from wild-type mean. Reference cell arbor sizes in filtered dataset are comparable between genotypes (wild-type *n* = 26; *Megf10* mutant *n* = 13). (L) Summary (as in C) of Sox2^+^ cell enclosure in P0 *Megf10* mutants and wild-type controls using the size-matched dataset (K). Sample sizes are as follows: real arbors as in (K); unmatched controls, *n* = 32 per arbor for wild-type (832 total), *n* = 20 per arbor for mutant (260 total). Statistics: two-tailed Mann-Whitney test. (M) Histogram of *Megf10*^−/−^ enclosure frequencies (as in D and E). Real mutant distribution (sample size as in K) was indistinguishable from chance rate (unmatched; *n* = 245). Statistics: chi-squared test. (N) Enclosed cell indices for individual P0 arbors (as in F) for size-matched dataset (left) and full dataset of mutant OFF reference cells (right; see F for full wild-type dataset). Sample sizes are as follows: left, as in (K) and (M); right, *n* = 29 real arbors, 20 unmatched images per arbor (zero values excluded, *n* = 479 total). Error bars, mean ± SD (J and K); mean ± SEM (D, E, and M) median ±95% confidence interval (F and N); min-max values (C and L). *p* values are shown on graphs; red text denotes value below alpha threshold. Scale bars, 10 μm. Also see Figure S3.

To test the first prediction, we marked individual OFF starburst cells using Chat:mGFP and measured how many Sox2^+^ starburst somata were enclosed within the reference cell’s dendritic territory. For each mGFP^+^ reference cell, we acquired confocal Z-stacks encompassing both IPL and INL dendritic arbors, drew a polygon demarcating the dendritic territory, and counted the number of Sox2^+^ nuclei fully contained within that polygon (Figure 3A). Measurements were made on the real image (*n* = 36 reference cells) as well as a family of “unmatched” images (*n* = 32 per reference cell) in which the reference cell dendritic outline was placed arbitrarily onto OFF starburst arrays from different retinal locations (Figure 3A). This unmatched control condition quantified the number of enclosed cells expected by chance in the absence of spatial coordination between dendrites and adjacent somata. At P0, >60% of real starburst arbors contained only a single cell body—the soma belonging to the reference Chat:mGFP^+^ cell (Figure 3D). By contrast, unmatched simulations showed significantly higher frequencies of multiple cell enclosure (Figure 3D) and enclosed more neighboring cells, on average, than real arbors (Figure 3C). We next performed a more stringent test, in which each real arbor’s enclosure rate was compared only to unmatched simulations from that same arbor. This test also showed that starburst somata are found within the reference cell arbor less often than expected by chance (Figure 3F). These findings support the conclusion that P0 starbursts avoid residing within neighboring cells’ dendritic territories (Figure 3G).

By P3, dendrites have grown larger (Figure S3A) and extend past the first ring of adjacent homotypic cells (Figure 3B). Accordingly, at P3, we found no difference in soma enclosure frequency between real and unmatched arbors, demonstrating that dendrites no longer exclude neighboring somata (Figures 3B–3F). Altogether, this analysis indicates that starbursts can exclude homotypic somata from their dendritic territories, but this occurs only during the time when exclusion zones are being established.

### MEGF10 enforces exclusion between OFF starburst dendrites and neighboring somata

If dendrite-soma exclusion is relevant for starburst mosaic formation, this exclusionary relationship should be disrupted in *Megf10* mutants. To test this idea, we investigated how deletion of MEGF10 affects the anatomy of early starburst homotypic contacts. At E16, *Megf10* mutant starbursts extended dendrites that aligned with neighboring somata, and frequently contacted them, in a manner that resembled control starbursts (Figures 2D, 2N–2P, S2G, and S2H). Thus, mutant starbursts are not deficient at establishing dendro-somatic homotypic contacts. However, by P0, we noticed that mutant somata were often located within neighboring dendritic territories (Figure 3I), raising the possibility that early embryonic contacts may not properly trigger dendrite-soma exclusion (Figures 3G and 3H, right panel).

To test whether P0 mutant starburst cells are indeed deficient in dendrite-soma exclusion, we measured the frequency of neighboring soma enclosure by *Megf10*^−/−^ Chat:mGFP^+^ OFF starburst dendrites. We found that real *Megf10*^−/−^ GFP^+^ dendrites enclosed starburst cell bodies at a similar frequency to unmatched controls (Figures 3N and S3C), suggesting that, in contrast to wild type, mutant somata are not excluded from dendrite territories. However, we also noted that *Megf10*^−/−^ arbors were smaller than wild-type arbors (Figure 3J), complicating efforts to compare enclosure rates between genotypes. To control for arbor size, we filtered wild-type and mutant reference cell datasets to include only arbors that were within 1 standard deviation of the wild-type mean size. This filtering generated a set of wild-type and mutant reference cells with comparable arbor sizes (Figure 3K). Using these size-matched datasets, we found that dendrite-soma exclusion was impaired in *Megf10* mutants, as mutant arbors enclosed significantly more somata than wild-type arbors (Figure 3L). Moreover, the number of enclosed cells within real mutant dendrites was indistinguishable from the chance rate measured from unmatched controls, suggesting that mutant arbors do not influence neighboring cell locations (Figures 3L–3N). Together, these findings demonstrate that dendrite-soma exclusion is absent in *Megf10* mutants, supporting the notion that such exclusion is the cellular mechanism underlying OFF starburst mosaic spacing.

To bolster evidence for this soma exclusion model, we also examined an alternative model of the *Megf10* mutant phenotype in which soma exclusion is unaffected. In order for soma positions to become random while still preserving dendrite-soma exclusion, the sizes of mutant arbors must necessarily become more variable than wild-type arbors (see illustration, Figure 3H). Contrary to this prediction, we did not detect differences in arbor size variability between wild-type and mutant starbursts (Levene’s variance test, f-ratio = 0.45, *p* = 0.51; note standard deviations in Figure 3J), arguing against the alternative model. Altogether, therefore, our evidence is in line with the conclusion that failure of dendro-somatic exclusion underlies the *Megf10* phenotype.

### MEGF10 functions as a repulsive transcellular signaling cue

How does MEGF10 enforce dendrite-soma exclusion? MEGF10 is a cell-surface protein that is expressed throughout the dendritic arbor and on the soma of embryonic and neonatal starburst neurons^4^ (Figures S2D and S2E). Thus, it is well positioned to send and receive transcellular signals initiated by starburst homotypic contacts—including dendrite-soma contacts. Indeed, MEGF10 can serve as both ligand and receptor in starburst neurons to mediate soma repulsion during mosaic patterning.^15^ Based on these prior results, we hypothesized that MEGF10 conveys a transcellular repulsive signal upon dendrite-soma contact, which prevents somata from settling in a neighboring cell’s dendritic territory. If this is true, then deletion of MEGF10 from a single starburst cell should prevent that cell from sending repulsive cues upon contact with its neighbors, thereby impairing its ability to exclude those neighbors from its exclusion zone.

**Figure 4.**
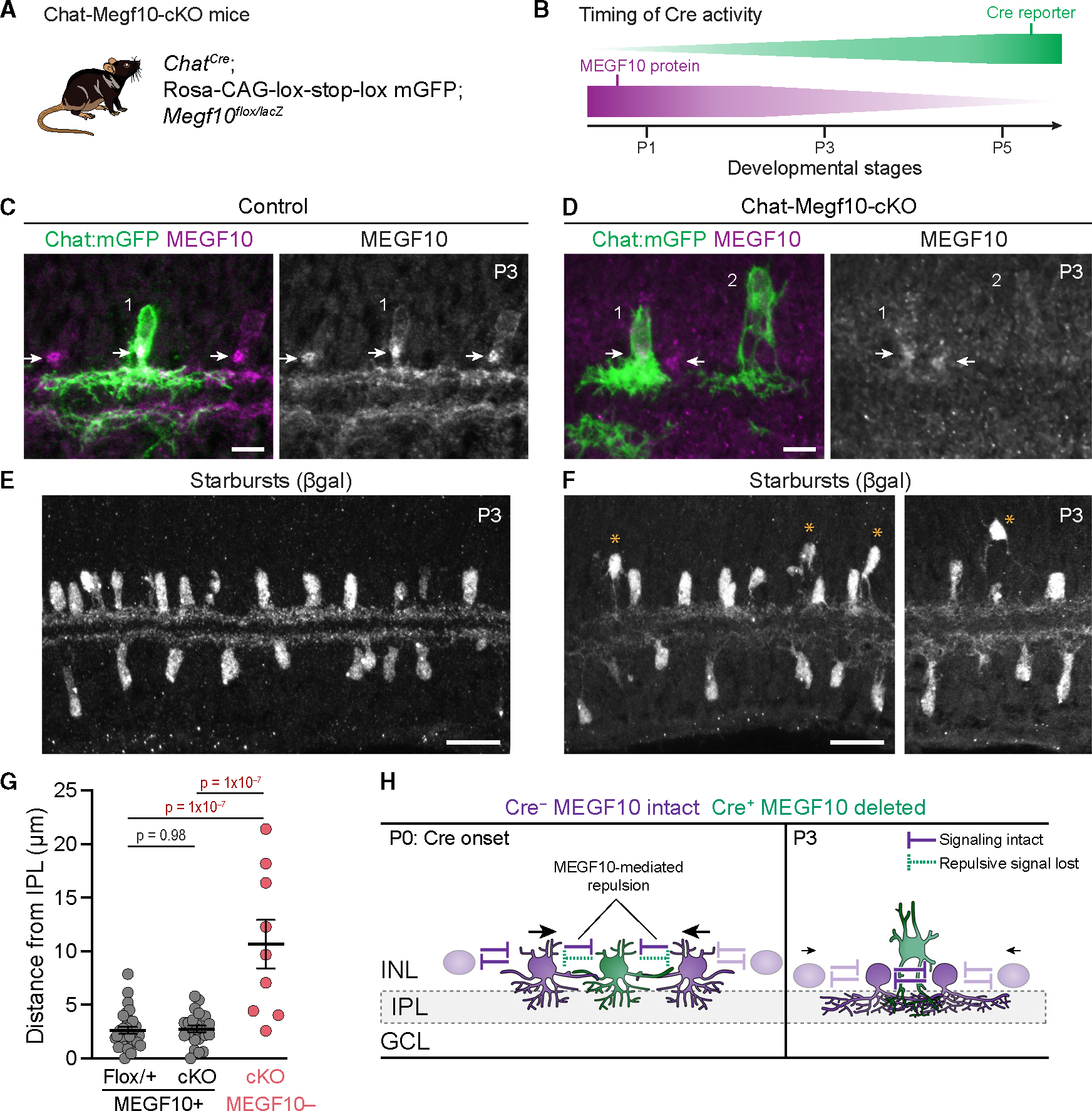
Starbursts lacking MEGF10 are impaired in repelling MEGF10^+^ neighbors. (A and B) Strategy for sparse deletion of MEGF10. (A) Genotype of mice. (B) Schematic summarizing time course of *Chat*^*Cre*^ activity and MEGF10 protein loss, as determined in our prior study.^4^ (C and D) Identification of MEGF10^−^ starburst cells at P3 using anti-MEGF10. Representative cross-sectional images of control retina (C, genotype *Chat*^*Cre*^; *Megf10*^*flox/+*^) and Chat-Megf10-cKO (D). Green, mGFP^+^ cells with early Cre activity. Anti-MEGF10 labels starburst somata and IPL arbors and is particularly strong at putative Golgi apparatus (arrows). In cKO retina (D), due to perdurance of MEGF10 protein, only a subset of mGFP^+^ cells are MEGF10^−^. Cell 1 is close to IPL and still expresses MEGF10 (arrow). Cell 2, which lacks MEGF10, is distant from IPL. (E and F) βgal immunostaining reveals full starburst population at P3. In controls (E, genotype *Megf10*^*lacZ/+*^), OFF starburst somata occupy a single INL stratum. In Chat-Megf10-cKO retina (F), a subset of OFF starbursts is located above the INL stratum where MEGF10^+^ starbursts reside. Asterisks indicate mutant cells located >10 μm from IPL, which is never observed for MEGF10^+^ starbursts (see G). (G) Quantification of mGFP^+^ OFF starburst soma distance to IPL at P3, using images similar to (C) and (D). Sample sizes are as follows: Flox/+, *n* = 29 cells; cKO MEGF10^+^, *n* = 24 cells; and cKO MEGF10^−^, *n* = 9 cells. Statistics: one-way ANOVA with post-hoc Tukey’s test (corrected for multiple comparisons). Error bars, mean ± SEM. For ON starbursts, see Figure S3E. (H) Working model based on results in (C)–(G) and Ray et al. and Kay et al.^4,15^ Starburst neurons engage in mutual MEGF10-mediated repulsion to establish mosaic. Onset of Cre in green cell (left panel) causes loss of repulsion, creating imbalance of forces onto adjacent cells (arrows). Adjacent cells are thereby pushed into mutant cell’s territory (right panel), excluding it from the INL stratum where mosaic is forming. Scale bars, 10 μm (C and D); 25 μm (E and F). Also see Figure S3.

To test this prediction, we leveraged the stochastic sparse activity of *Chat*^*Cre*^ at neonatal stages (Figures 2D–2F) to achieve sparse deletion of MEGF10 from individual starburst cells. A *Megf10*^*flox*^ conditional mutant allele was bred with *Chat*^*Cre*^, generating Chat-Megf10-conditional knockout (cKO) mice (Figure 4A). In a previous study,^4^ we characterized MEGF10 protein expression dynamics in these Chat-Megf10-cKO mice (Figure 4B). Due to the timing of *Chat*^*Cre*^ expression, as well as perdurance of MEGF10 protein following Cre onset, MEGF10 is not eliminated from the starburst population until P5.^4^ At P3, most Chat-Megf10-cKO starbursts still express MEGF10 at sufficient levels to prevent mutant phenotypes^4^ (Figures 4C, 4D, and 4G). However, a small number of P3 starbursts already lack MEGF10 protein^4^ (Figures 4B–4D), indicating that they were subject to earlier Cre activity than the rest of the population and therefore lost MEGF10 during the key period prior to P3, when dendrite-soma exclusion occurs (Figure 3). Importantly, MEGF10^−^ cells were only a small minority of the P3 starburst population, such that they were typically surrounded by MEGF10^+^ neighbors. Here, we studied these MEGF10^−^ cells, and control MEGF10^+^ cells from the same cKO retinas, to ascertain the consequences of losing the ability to send MEGF10-mediated signals. The analysis was performed in cross-sections, as the MEGF10 antibody staining required to identify MEGF10^−^ cells was not reliable in whole mounts.

Comparison of cKO starbursts that contained or lacked MEGF10 revealed a striking difference in soma positioning: whereas MEGF10^+^ OFF cells were aligned in a single plane, close to the IPL, MEGF10^−^ cells were often located farther from the IPL, at a different INL level than their MEGF10^+^ neighbors (Figures 4C–4G). This phenotype suggests that MEGF10^−^ cells are not incorporated into the 2D plane in which the OFF starburst mosaic is forming. A similar effect on soma positioning was seen for cKO ON cells lacking MEGF10 (Figure S3E). By contrast, in constitutive *Megf10* mutants, starbursts remained well aligned in a single 2D plane,^4^ suggesting that the cKO positioning phenotype arises due to interactions between mutant and wild-type cells. Some cKO MEGF10^−^ cells were located directly above normally positioned MEGF10^+^ cells, suggesting that mutant cells are unable to prevent neighboring MEGF10^+^ cells from entering their territory (Figure 4F; see model; Figure 4H). Together, these results support the conclusion that MEGF10 mediates transcellular homotypic signals during establishment of starburst exclusion zones, such that cells without MEGF10 cannot repel their MEGF10^+^ neighbors, leaving them at a disadvantage in claiming territory within the 2D mosaic plane (Figure 4H).

### Starburst tangential movements occur normally in *Megf10*^−/−^ retina

We next addressed the cellular mechanisms leading to loss of starburst exclusion zones when MEGF10-mediated repulsion is absent. Lateral soma movements within the tangential plane of the retina are thought to reposition neurons from the site of their birth, which is random, into an orderly mosaic array.^12,14,17–19^ We therefore examined how MEGF10 influences these tangential movements. One possibility is that *Megf10*^−/−^ starbursts might be unable to move from the site of their birth. In this case, repulsive contacts with neighboring dendrites would not have an opportunity to influence soma position. Alternatively, mutant starbursts may still move but without constraints imposed by dendrite contact, such that they can enter neighboring arbor territories.

**Figure 5.**
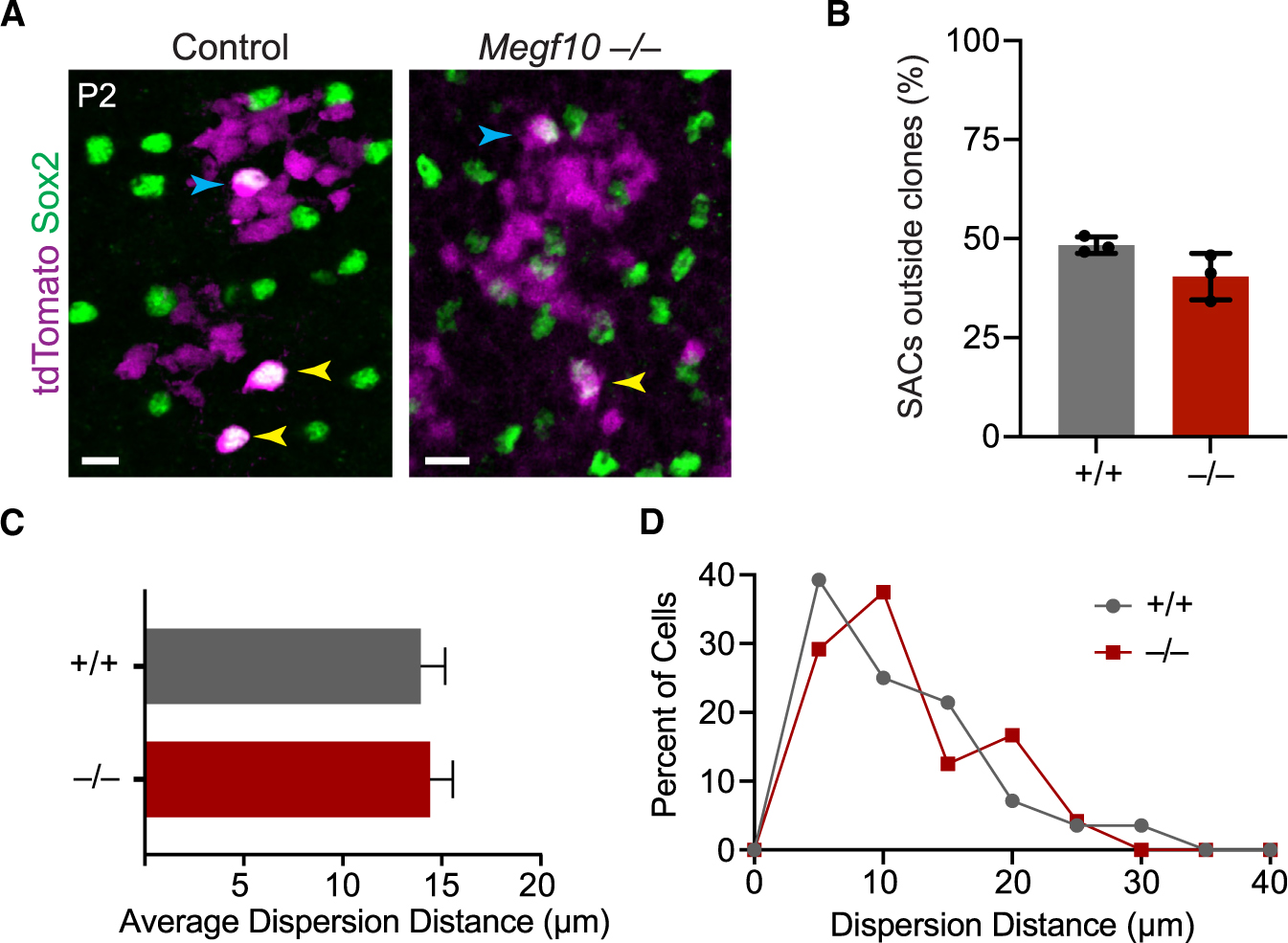
Starburst tangential movements are normal in *Megf10* mutants. (A) *En face* views of INL in Pax2-Cre; tdTomato reporter mice; tdTomato labels clonally related columns of neurons. Sox2^+^ starburst cells are detected within clonal columns (blue arrows) and also outside of columns indicating tangential dispersion (yellow arrows). OFF starbursts move tangentially both in *Megf10*^*+/+*^ controls (left) and *Megf10*^−/−^ mutants (right). Scale bar, 10μm. (B) Frequency of tangential migration, measured as percentage of tdTomato^+^ starbursts outside clonal columns. Sample sizes are as follows: *n* = 3 animals per genotype, 50–60 cells per animal (wild-type, 196 cells total; mutant, 163 cells total). Statistics: two-tailed t test, *p* = 0.09. (C and D) Dispersion distance for starbursts outside clonal columns was unchanged in *Megf10* mutants (C, two-tailed t test, *p* = 0.78; *n* = 24 wild-type cells, *n* = 28 mutant cells). Distribution of dispersion distances was also unchanged (D). Error bars, SD.

To distinguish between these models, we tested whether *Megf10*^−/−^ starbursts are capable of tangential movements. Using a well-established approach,^17,29^ the extent of tangential dispersion was evaluated by marking clones of cells derived from a small subset of retinal progenitors and then measuring the lateral displacement of starburst progeny from their clone of origin. A Pax2-Cre BAC transgenic line,^30^ crossed to a tdTomato Cre reporter, was used to mark progenitors. In control P2 retina, densely packed radial clones of Tomato^+^ neurons were distributed across the retina (Figure 5A). The Tomato reporter also labeled laterally displaced individual neurons that had moved away from their clone of origin via tangential migration—some of which were Sox2^+^ starburst neurons (Figure 5A). Other Tomato^+^ Sox2^+^ starbursts remained aligned with Tomato^+^ clones, indicating that they either did not migrate or settled at the site of a different labeled clone (Figure 5A). To evaluate tangential migration in *Megf10* mutants, we quantified the fraction of starbursts that were not aligned with labeled clones—i.e., those that had definitively migrated—as well as the distance migrated by displaced cells. No difference between mutant and wild type was detected on either measure (Figures 5B–5D). Thus, starbursts move tangentially even without *Megf10*, implying that the mutant phenotype arises because neighboring dendrites are unable to prevent motile starbursts from moving into their arbor territories.

### Dendrite-soma exclusion also patterns the ON starburst mosaic

**Figure 6.**
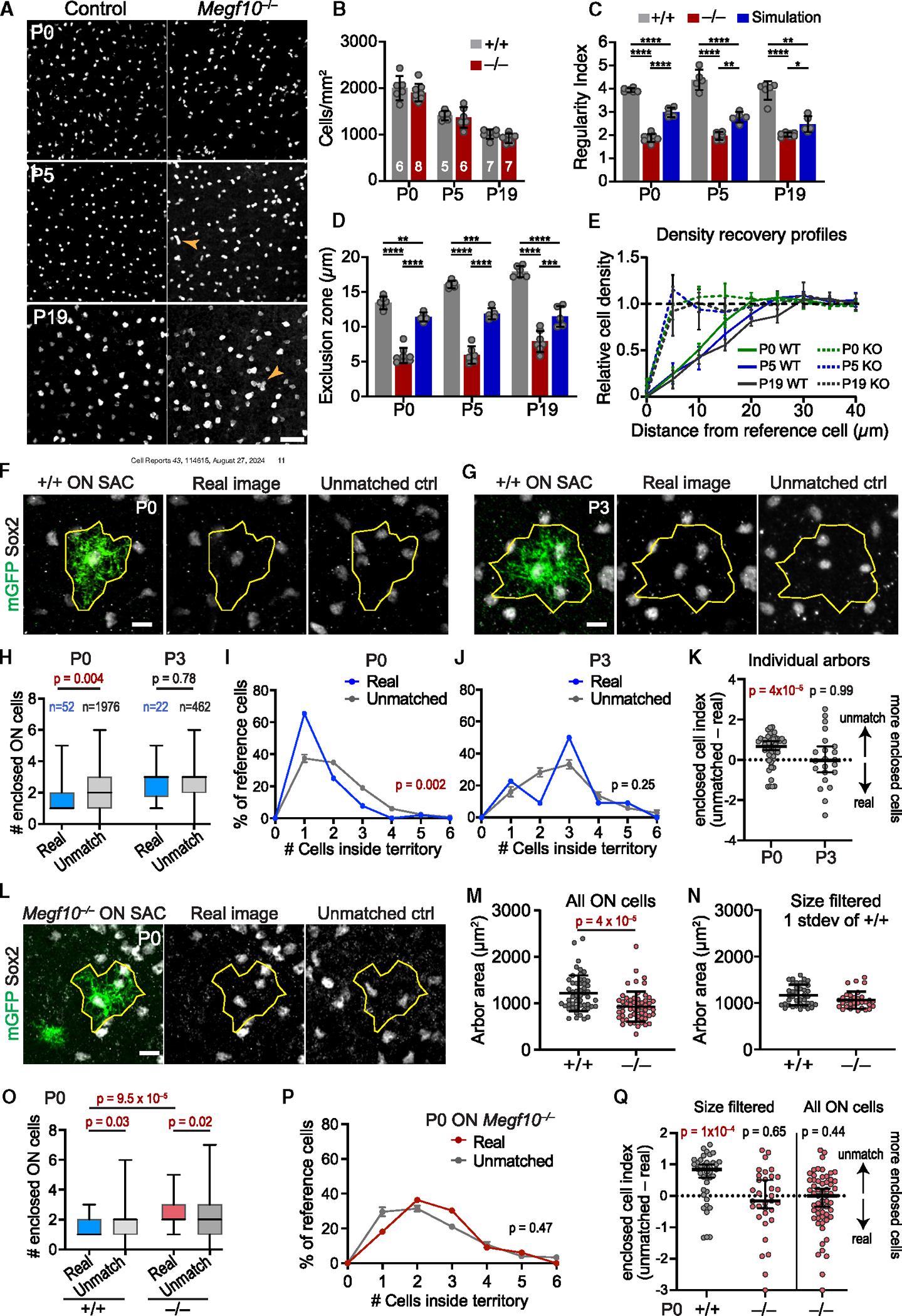
MEGF10 mediates ON starburst mosaic spacing and dendrite-soma exclusion. (A) Representative images of ON starburst array across development in wild-type controls and *Megf10* mutants. Cells were labeled with anti-Sox2 (P0 and P5) or anti-ChAT (P17). Arrowheads, small starburst clumps. (B) ON starburst density did not differ between genotypes (two-way ANOVA, no main effect of genotype, *p* = 0.18). (C) ON starburst regularity, assessed by VDRI (see Figure S1), was lower in mutants than in controls. Mutant regularity was also lower than for simulated random arrays. Statistics: 1-way ANOVA (main effect at all ages, *p* > 1 × 10^−7^) followed by Tukey’s post-hoc test, *p < 0.05, **p < 0.01, ****p < 1 × 10^−5^. (D and E) Exclusion zone size (D), measured using DRP (E), increased over time in wild-type mice (one-way ANOVA to test for age effects in wild-type group only, main effect of age *p* =4 × 10^−7^). Statistics (D): 1-way ANOVAs were used to test for genotype effects at each age (main effect of genotype at all ages, *p* >1 × 10^−7^) with Tukey’s post-hoc test, **p < 0.01; ***p < 0.001; ****p < 1 × 10^−5^. (F and G) Representative Chat:mGFP^+^ ON starburst reference cells from P0 (F) or P3 (G) wild type (+/+), shown with their real Sox2^+^ starburst arrays (left and center) and with unmatched ON arrays (right). Yellow, dendritic polygon. (H) Summary of ON Sox2^+^ cells fully enclosed by real dendrites (blue) or unmatched controls (gray). Sample sizes are as follows: P0, *n* = 52 real arbors from 7 animals; *n* = 1,976 control images (38 unmatched images per arbor); P3, *n* = 22 real arbors; *n* = 462 control images (21 unmatched images per arbor). Statistics: two-tailed Mann-Whitney test. (I and J) Fraction of ON reference cells enclosing *n* somata at P0 (D) or P3 (E). Sample sizes are as follows: real arbors as in (C); unmatched controls (zero values excluded), P0, *n* = 1,827; P3, *n* = 457. Statistics: chi-squared test. (K) Individual P0 arbors enclose fewer Sox2^+^ cells than their own unmatched controls, as shown by the enclosed cell index. P3 arbors enclosed Sox2^+^ cells at the chance rate (dashed line). Sample sizes are as in (H). Statistics: Wilcoxon one-sample test with theoretical median of 0. (L) Representative ON Chat:mGFP^+^ reference cell from P0 *Megf10*^−/−^ retina, shown with its real Sox2^+^ starburst array (center) and with an unmatched ON starburst array (right). (M) ON starburst arbor size is smaller in *Megf10* mutants than in controls (wild type, *n* = 52 as in (B); mutant, *n* = 58 cells from 7 animals; two-tailed t test). (N) Filtering of arbors >1 standard deviation from wild-type mean. Reference cell arbor sizes in filtered dataset are comparable between genotypes (wild type, *n* = 39; mutant; *n* = 33). (O) Plot (as in H) summarizing P0 Sox2^+^ cell enclosure in *Megf10* mutants and wild-type controls using the size-matched dataset (N). Sample sizes are as follows: real arbors as in (N); unmatched images: wild type, *n* = 38 per arbor (1,482 total); mutant, *n* = 32 per arbor (1,056 total). (P) Histogram of ON *Megf10*^−/−^ enclosure frequencies (as in I and J). Real mutant distribution (sample size as in N) was indistinguishable from chance distribution (unmatched; *n* = 906). Statistics: chi-squared test. (Q) Enclosed cell indices for individual ON P0 arbors (as in K) for size-matched dataset (left) and full dataset of mutant ON reference cells (right; see K for full wild-type dataset). Sample sizes are as follows: left, as in M and N; right, *n* = 58 real arbors, 32 unmatched images per arbor (zero values excluded, *n* = 1,430 total controls). Statistics are as in (K). Error bars, mean ± SD (B–E, M, and N); mean ± SEM (I, J, and P); median ±95% confidence interval (K and Q); min-max values (H and O). *p* values are shown on graphs; red text denotes value below alpha threshold. Scale bars, 50 μm (A); 10 μm (F, G, and L). Also see Figure S3.

To learn whether other cell types also use dendrite-soma exclusion for mosaic patterning, we investigated development of the ON starburst mosaic. Similar to OFF cells (Figure 1), wild-type ON starbursts were already regularly spaced at P0 (Figures 6A–6E). By contrast, in *Megf10* mutants, the ON array lacked exclusion zones at P0 (Figure 6D) and regularity failed to improve with age (Figures 6C–6E). To test whether dendrite-soma exclusion contributes to ON starburst patterning, we analyzed the spatial relationship between single Chat:mGFP^+^ arbors and neighboring ON somata, measuring the chance rate of enclosure using the unmatched control methodology (Figures 6F and 6G). At P0, but not at P3, real ON starburst arbors contained fewer neighboring somata than expected by chance, with the majority (>60%) of arbors enclosing only the reference Chat:mGFP^+^ soma (Figures 6F–6K). The exclusionary relationship between P0 dendrites and neighboring somata was lost in *Megf10* mutants (Figures 6L–6Q). These results indicate that ON and OFF starburst mosaics develop similarly: in both cases, dendrites repel neighboring somata in a *Megf10*-dependent manner to establish exclusion zones prior to P3.

**Figure 7.**
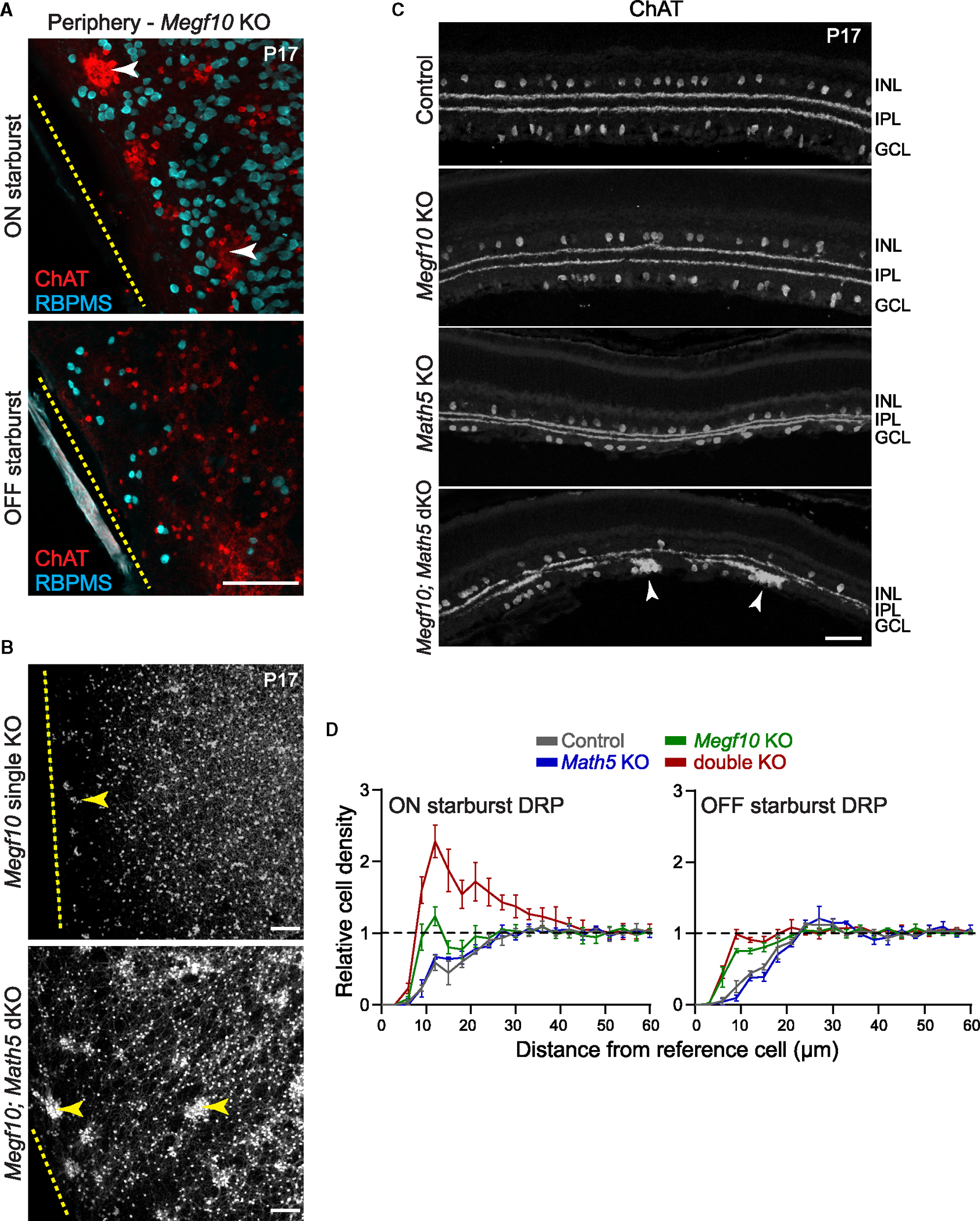
ON starburst aggregation in the absence of MEGF10 and retinal ganglion cells. (A) Representative *en face* images of *Megf10*^−/−^ P17 retinal whole mount at far periphery. Anti-ChAT (starbursts); anti-RBPMS (RGC somata). Images are from a single z stack at level of GCL (top) or INL (bottom). Note ON starburst clumps (arrowheads) adjacent to retinal edge (dashed line). (B) Lower-magnification images of peripheral retina (as in A). Multicellular aggregates (arrows) are larger and more widespread in *Megf10; Math5* dKO than in *Megf10* single KO. Dashed line, retinal edge. (C) P17 cross-sections through central retina; starbursts are labeled with anti-ChAT. Soma aggregates in *Megf10* single KOs are limited to ~3 cell bodies. In *Megf10; Math5* dKOs, ON starburst aggregates are larger (arrowheads). Images are representative of at least two different animals. (D) Quantification of P17 ON and OFF starburst aggregation/repulsion using density recovery profiles (DRPs; Figure S1). Annulus size, 3 μm. For wild-type and *Math5* single mutants, starburst density is lower at short intercellular distances than global cell density (dashed line), demonstrating local cell-cell avoidance (i.e., exclusion zones). In dKOs, ON cell density is higher at short spatial scales than global density, demonstrating cell aggregation. *Megf10* single KOs also show mild ON cell aggregation but far less than dKOs. OFF starbursts do not aggregate. Error bars, SD. Scale bars, 100 μm (A and B); 50 μm (C). Also see Figure S1.

While the fundamental exclusion zone mechanism appears quite similar for OFF and ON starbursts, our analysis did uncover one notable difference between the two cell types: whereas OFF starbursts are arranged randomly in *Megf10* mutants (Figures 1E and 1F), we observed that loss of *Megf10* unveils an attractive interaction among ON starburst cells. Two lines of evidence support this finding. First, ON *Megf10*^−/−^ exclusion zone sizes were smaller, and mosaic regularity was lower, than would be expected for an array of randomly distributed cells (Figures 6C and 6D). Thus, mutant ON arrays are not in fact random but instead display mild aggregation. Second, we noted anatomical examples of ON (but not OFF) starburst clustering in *Megf10* mutants (Figure 6A). Large clumps were particularly notable at P17 in far peripheral retina (Figure 7A), within regions that were outside the areas used for spatial statistical analysis shown in Figure 6. Together, these results indicate that loss of MEGF10-mediated repulsion has distinct effects upon the OFF and ON arrays: OFF starbursts become randomly positioned, whereas ON starbursts tend to attract each other.

### Retinal ganglion cells influence ON starburst mosaic patterning

Finally, we investigated the origins of ON starburst aggregation in *Megf10* mutants. The incidence and size of starburst clumps was largest at the far retinal periphery (Figure 7A), suggesting that factors promoting homotypic attraction are most prevalent in peripheral retina. One factor that varies along the center-peripheral axis is density of retinal ganglion cells (RGCs), the largest cell population residing within the GCL alongside ON starburst cells. At the far periphery, RGC density is over 2-fold lower than in central retina.^31,32^ By contrast, ON starburst density declines only minimally (~1.33-fold) between center and periphery.^25^ Therefore, starburst cells comprise a larger fraction of GCL neurons in the periphery, where clumping is prominent, than in central regions where clumping is rare. As such, ON starburst somata are more likely to interact directly with each other, rather than interacting with an RGC, in the far periphery where clumping phenotypes are strongest. This observation suggests that heterotypic interactions with RGCs might influence ON starburst aggregation by shielding them from homotypic attractive interactions.

If this model is correct, the likelihood of clumping in *Megf10* mutants is directly related to the starburst:RGC ratio. Thus, increasing the starburst:RGC ratio by genetic removal of RGCs should enhance the *Megf10*^−/−^ aggregation phenotype. This prediction was tested by deletion of the Math5 transcription factor, which induces a near-complete absence of RGCs^33–35^ but does not impact differentiation of starburst neurons^36^ (Figure 7C). In *Megf10*^−/−^*; Math5*^−/−^ double knockout (dKO) mice, starburst density was similar to wild-type controls (OFF WT: 1,495 ± 107.4 cells/mm^2^; OFF dKO: 1,648 ± 102.8 cells/mm^2^; ON WT: 1,290 ± 39.8 cells/mm^2^; ON dKO: 1,310 ± 8.1 cells/mm^2^; mean ± SEM, *n* = 3 animals per genotype). However, DRP spatial analysis revealed that ON starburst aggregation was markedly increased in dKOs relative to wild-type or to *Megf10* single mutants (Figure 7D). This effect was specific to ON starbursts (Figure 7D). In contrast to *Megf10* single mutants, which only exhibited strong clumping at the extreme periphery, numerous clumps were present throughout the dKO retina (Figures 7B and 7C). Furthermore, starburst aggregates were typically much larger in dKOs than in *Megf10* single mutants (Figures 7B–7D). These results demonstrate that absence of RGCs increases the frequency of attractive interactions among ON starburst neurons. Importantly, starburst exclusion zones were normal in *Math5* single mutants, indicating that homotypic repulsion was intact (Figures 7C and 7D). Thus, MEGF10-mediated repulsion is sufficient to overcome any enhanced attraction resulting from removal of RGCs. Together, these findings suggest that ON starbursts possess an intrinsic homotypic attractive activity, which is countered both by MEGF10 repulsion and by the presence of heterotypic neurons, which serve to buffer starburst cells from interacting and adhering to each other.

## DISCUSSION

Establishment of retinal mosaics involves dendritic contacts among homotypic neurons, which generate a repulsive signal that establishes exclusion zones.^12^ In this study, we clarify the mechanism by which dendritic contacts create exclusion zones among starburst amacrine cells. The prevailing model has been that homotypic contacts occur at dendritic tips, leading to transient dendrite tiling that carves out a unique territory for each cell^6,7,21^ (see schematic, Figure 2B). To the contrary, however, we show here that the key repulsive interaction occurs between nascent dendritic arbors and neighboring starburst somata. Dendro-somatic repulsion provides a compelling explanation for one of our key findings: that starburst cell bodies are excluded from their neighbors’ dendritic territories during the E16–P0 period. The most parsimonious explanation for this exclusionary relationship is that starburst dendrites and somata can repel each other. Supporting this idea, we show that dendrite-soma contacts are extensive at E16–P0, providing an anatomical basis for repulsive interactions leading to dendrite-soma exclusion. Finally, using *Megf10* mutants, we provide evidence that dendrite-soma repulsion is required for exclusion zone formation and mosaic patterning. Thus, we conclude that dendrite-soma-repulsive contacts are well positioned to serve as the cellular mechanism for establishing starburst mosaics.

The molecular mechanism underlying starburst dendrite-soma repulsion is likely initiated by MEGF10 transcellular signaling. This conclusion is supported by our current data as well as our prior work showing that MEGF10-mediated repulsion is necessary and sufficient for establishing starburst exclusion zones.^4,15^ The precise nature of the cell-cell contacts triggering repulsive MEGF10 signals had until now remained unclear, but our present results support the view that they occur at dendrite-soma contact sites. Here, we show that dendrite-soma contacts still occur in *Megf10* mutants, but they no longer impart a repulsive signal, as P0 mutant arbors do not exclude neighboring somata. Furthermore, analysis of individual mutant cells shows that MEGF10 transmits an intercellular repulsive signal that prevents encroachment from neighboring starbursts. Altogether, the data support a model whereby dendrite-soma contact triggers bidirectional repulsive MEGF10-mediated signals, which prevent dendrite-soma overlap, leading to establishment of exclusion zones.

How might this dendrite-soma exclusion mechanism be implemented to pattern mosaics? Mouse starburst cells are born over a period of several days during mid-gestation.^37,38^ During this time, new cells completing their radial migration are continually added to an established mosaic that is already regularly spaced.^14^ Therefore, models of mosaic formation must account for sequential addition and constant refinement of the mosaic as new cells are added. Based on our present results, we propose the following working model (illustrated in the graphical abstract).

As a newborn starburst neuron arrives at the embryonic inner retina, it initiates arbor growth toward somata of other starbursts that arrived earlier.^4^ Initial contacts may occur within the nascent IPL and/or within the soma layers, but in either case, the result is similar: When dendrites of the newborn cell contact the soma of a pre-existing neighbor, a repulsive signal mediated by MEGF10 is transmitted within both cells. For the dendrites of the newborn cell, this signal restrains further growth, while for the cell bodies of the pre-existing cells, the signal initiates tangential movement to make space for the new cell within the mosaic. Each pre-existing cell then adjusts its position until the MEGF10-mediated repulsive force on all sides is in equilibrium—a mechanism that is plausible based on our prior MEGF10 misexpression studies^15^ (see schematic, Figure 4H). These movements account for at least some aspects of embryonic starburst tangential motility, although since *Megf10*^−/−^ starbursts are still motile, there must be other forces encouraging tangential movement. As surrounding cells move away, the new cell is able to further extend its arbors, thereby repeating the cycle of repulsive signaling and cell movement. In this way, the newborn starburst cell carves out an exclusion zone for itself that is defined by the extent of its dendritic arborization.

There is precedent for the idea that regular spacing of retinal neurons could involve exclusion of homotypic somata from dendrite territories. In adult primate retina, parasol cells exhibit dendrite-soma arrangements that are strikingly similar to the arrangements of developing starburst neurons: parasol somata in the GCL are precisely aligned over the edges of their neighbors’ IPL dendritic territories.^22^ This alignment is maintained despite large variations in cell density and dendritic size across different retinal eccentricities,^22^ which suggests that alignment results from dendrite-soma coordination during development. Mouse and primate horizontal cells also show similar dendrite-soma coordination, which is preserved as arbor size and cell density are varied.^7,23^ Several other potential examples of dendrite-soma alignment include multiple mouse RGC types within the online “RGC museum,”^39^ mouse Vglut3^+^ amacrine cells,^40^ and several types of melanopsin-expressing RGCs.^41^ Thus, dendrite-soma alignment appears to be widespread, in which case, dendrite-soma repulsion could be involved in mosaic patterning of many retinal cell types beyond starburst neurons. This conclusion does not rule out the possibility of other patterning mechanisms: while we did not find evidence that starbursts tile, tiling may still mediate exclusion zone formation for other cell types that do tile, such as primate midget RGCs^42^ and mouse horizontal cells.^21^

### Limitations of the study

Several questions about MEGF10 and its role in exclusion zone formation remain unresolved by our study. First, what is the nature of the tangential movements driving exclusion zone formation? These movements could be characterized by live imaging of starbursts within retinal explants; this is presently beyond our capabilities but could be included in a future study. Second, while we show that *Megf10*^−/−^ starburst arbors cannot exclude neighboring somata, we have not delineated mechanisms downstream of MEGF10 that mediate this repulsion. MEGF10 and its invertebrate homologs, Draper and CED-1, are known regulators of the actin cytoskeleton^43,44^; thus, cytoskeletal properties of mutant arbors may be altered in a manner that diminishes their ability to resist entry by neighboring cells. Future experiments should test this model. Finally, we cannot yet explain why MEGF10-mediated homotypic repulsion is specific to dendrite-soma interactions. In principle, dendro-dendritic contacts should also trigger MEGF10 repulsion, since starburst dendrites express MEGF10 and overlap extensively. However, starburst dendrites exhibit homotypic adhesion rather than repulsion. One plausible explanation is that starburst dendrites have been shown to express a variety of adhesion molecules that are not detected immunohistochemically on the soma.^45–47^ These dendrite-specific adhesion molecules may overcome MEGF10-mediated repulsion upon dendrite contact, whereas their absence from somata may enable MEGF10 repulsion to dominate when soma contact occurs. Further work will be needed to test this model.

Altogether, our study highlights cellular mechanisms that contribute to mosaic patterning for ON and OFF starburst cells and which have the potential to pattern many other cell types as well. Future studies will reveal the extent to which this mechanism, as opposed to tiling or other modes of homotypic interaction, may explain exclusion zone formation by other retinal cell types.

## STAR★METHODS

### RESOURCE AVAILABILITY

#### Lead contact

Requests for resources and reagents should be directed to and will be fulfilled by the lead contact, Jeremy Kay (jeremy.kay@duke.edu).

#### Materials availability

*Megf10*^*Cre*^ mice generated in this study are available from the lead contact upon request; ultimately we expect they will be available from one of the major mouse repositories, as we are actively working to deposit them at the time of writing. The *Megf10*^*CreNeo*^ targeting construct is also available from the lead contact upon request.

#### Data and code availability

- All data reported in this paper will be shared by the lead author upon request.
- This paper does not report original code.
- Any additional information required to reanalyze the data reported in this paper is available from the lead contact upon request.

### EXPERIMENTAL MODEL AND STUDY PARTICIPANT DETAILS

#### Mice

Animal experiments were reviewed and approved by the Institutional Animal Care and Use Committee of Duke University. Mice of both sexes were used. The animals were maintained under a 12 h light-dark cycle with *ad lib* access to food and water. Animals of both sexes were used in this study. Wild-type CD1 mice were purchased from Charles River.

For these studies, we used several existing transgenic and mutant mouse lines: (1) *Megf10*^*tm1b(KOMP)Jrs*^, referred to as *Megf10* KO, *Megf10*^*−*^ or *Megf10*^*lacZ*^, in which the *Megf10* locus drives expression of a *lacZ* reporter instead of the endogenous MEGF10 protein^15^; (2) *Megf10*^*tm1c(KOMP)Jrs*^, referred to as *Megf10*^*flox*^, used as a conditional loss-of-function allele^4^ (3) *Chat*^*tm2(cre)Lowl*^ (RRID:IMSR_JAX:006410),^51^ referred to as *Chat*^*Cre*^; (4) A germline Flp deleter strain, Tg(ACTFLPe)9205Dym/J (RRID:IMSR_JAX:005703); (5) Tg(Pax2-cre)1Akg/Mmnc (RRID:MMRRC_010569-UNC), known as Pax2-Cre^30^; (6) *Atoh7*^*tm1Gla*^ (RRID:MMRRC_042298-UCD), also known as *Math5*^*−*^ or *Math5* KO.^33^ We additionally used three different Cre reporter strains. Two of these strains express membrane-targeted green fluorescent protein (mGFP) upon Cre recombination: (1) *Gt(ROSA)26*^*Sortm4(ACTB-tdTomato,-EGFP)Luo*^ (RRID:IMSR_JAX:007576), also known as mT/mG^52^; (2) Gt(ROSA)26Sor^tm1(CAG–EGFP)Blh^ (MGI:3850169),^48^ also known as *Rosa26*^*fGFP*^. The third Cre reporter strain expresses tdTomato fluorescent protein upon Cre recombination: *Gt(ROSA)26Sor*^*tm14(CAG-tdTomato)Hze*^ (RRID:IMSR_JAX:007914).^53^

Mouse lines were obtained from Jackson Laboratories except the *Megf10* null mutant strain, which we previously generated; the *Math5* null mutant strain (kind gift of Tom Glaser, UC Davis); the *Rosa26*^*fGFP*^ strain (kind gift of Brigid Hogan, Duke University); and the Pax2-Cre strain (kind gift of Joshua Weiner, University of Iowa). The *Tm1a* allele of *Megf10*, which can be converted to the null allele using a germline Cre deleter strain or to the flox allele using a germline Flp deleter strain, is available from the Mutant Mouse Regional Resource Center (cat# MMRRC:068040-UNC).

In addition to these existing strains, we also generated new *Megf10*^*Cre*^ strains; specifics of how they were generated are provided in the Method Details section below.

### METHOD DETAILS

#### Generation of *Megf10* driven cre mouse lines

*Megf10*^*Cre*^ knock-in mouse lines were generated in collaboration with Duke Transgenic Core. The *Megf10* locus was targeted using homologous recombination and a modified pL253 vector^49^ with mc1-driven thymidine kinase for ES cell selection. Targeted ES cell clones were validated by PCR and Southern blot analysis before embryo injection. Founder mouse lines were validated by PCR. The targeting construct was engineered to modify exon 25 of *Megf10*, replacing the endogenous stop codon with an FLAG epitope tag, followed by a T2A self-cleaving peptide,^54^ to release a Myc-epitope tagged Cre recombinase (Figure S2A). The founder mouse line, *Megf10*^*CreNeo*^, contained an FRT-flanked neomycin resistance sequence downstream of the Cre. To generate the *Megf10*^*Cre*^ allele, the neomycin cassette was removed by crossing *Megf10*^*CreNeo*^ with a mouse line expressing germline FLP recombinase (see Animals section above for strain details). Removal of the neomycin resistance cassette was confirmed by PCR. During the coronavirus pandemic of 2020, due to severe constraints on mouse husbandry and staffing, we lost several mouse strains from our colony including the parental *Megf10*^*CreNeo*^ strain. However, the *Megf10*^*Cre*^ strain was not lost, and the targeting vector is still available in case the parental strain needs to be recreated.

#### Mosaic analysis

Using confocal microscopy, 40x images (353.55 × 353.55 μm) were acquired from whole-mount retinal preparations in mid-peripheral retina, using Olympus FV300 or FV3000 confocal microscopes. Z-stacks encompassed most of the inner retina, from the vitreal retinal surface to the middle of the INL. OFF and ON starburst populations were identified at different optical planes of these Z-stacks based on cell type-specific marker expression and their location in INL or GCL. Hoeschst counterstaining allowed identification of retinal layers within the Z-stacks. For mosaic analysis of developing OFF and ON starbursts (P0 and P5) we used anti-Sox2 as a cell type-specific nuclear marker.^24^ Sox2 is also expressed by nerve fiber layer astrocytes and Müller glia, but these could easily be distinguished from starburst neurons based on laminar location and/or nuclear morphology. For later timepoints (i.e., P19), anti-ChAT was used to label ON and OFF starburst populations. At E16, Sox2^+^ OFF and ON starbursts were intermingled due to ongoing radial migration. As a result, we were unable to distinguish the two starburst populations which precluded analysis of mosaic regularity or exclusion zone sizes at E16.

For measurements of cell size at P0 and P5, starbursts were labeled using antibodies to β-galactosidase in *Megf10*^*lacZ*^ reporter mice.^4,15^ This marker was preferable to Sox2 for size measurements because it filled the cytoplasm. At P19, ChAT was used to measure cell size as described previously.^15^ Sizes were measured in ImageJ by encircling cells with an ROI; the Feret’s diameter tool was then used to measure the maximum diameter of the ROI. Sample sizes for diameter measurements were at least *n* = 100 cells from at least two animals. At all ages, mean soma size was 10.0 μm so we used this value for all downstream analysis steps.

For analysis of spatial statistics, images were loaded into FIJI/ImageJ software^50^ and the point selector tool was used to manually mark the center of each starburst cell, generating X-Y coordinates. These coordinates were then used to produce Voronoi Domain Regularity Indices (VDRI) using Fiji software, and Density Recovery Profiles using WinDRP software as previously described^8,15^ (see Figure S1 for an illustration of these methods). Exclusion zone sizes were calculated from the DRP in WinDRP software, which implements Rodieck’s definition of the effective radius (i.e., exclusion zone) as the midpoint of the rising part of the DRP curve.^27^ Annulus size increment for DRP analysis was 5 μm.

Random simulations were generated using a custom MATLAB script as previously described.^55^ Briefly, the algorithm placed cells into a square field of view matching the size of the ones used for real images (i.e., 353.55 μm on all sides). The cells were placed one-by-one according to a Poisson point process, until the density of the array was equal to the mean starburst density measured from real data (Figure 1C). The only constraint on cell location was that two cells could not occupy the same physical location. To determine if two cells overlapped, each cell was assigned a diameter of 10 μm (the average cell size measured for real starburst neurons at all ages analyzed – see measurement details above). If a new cell was added to the array at a location where its diameter overlapped with a pre-existing cell, placement at that location was canceled and a new location was assigned based on the Poisson point process.

For experiments examining *Math5; Megf10* double knockout retinas, mosaic analysis was performed using 20x images (636.5 × 636.5μm) and the annulus step size used for DRP analysis was 3 μm.

#### Arbor territory analysis

The relationship between starburst dendritic arbor territories and neighboring homotypic somata was assessed using two channel images that contained 1) individually labeled starburst arbors from *Chat*^*Cre*^; mGFP reporter mice; and 2) co-staining with anti-Sox2 to label the nuclei of the entire ON and OFF starburst populations. Z-stacks encompassing the entire dendritic arbor, including both soma layer and IPL projections, and relevant nuclear layer were collected using confocal microscopy (Olympus FV300 or FV3000). For this imaging, we avoided far peripheral retinal regions – approximately one field of view away from the edge of the retina – that contained starburst clumps in *Megf10* mutants (Figure 7A). Dendrite territories were drawn by hand in ImageJ by drawing lines connecting arbor tips. This was done for each cell to generate a series of polygonal regions of interest (ROIs) for each mGFP^+^ starburst in the dataset. We counted the number of Sox2^+^ homotypic somata completely contained within those territories, including the soma belonging to the reference cell.

To assess if starburst arbor territories contained fewer homotypic somata than expected by chance, we performed these dendritic Sox2 counts on two groups of images. First, as the experimental group, we used real images, in which the dendritic ROI was overlaid over the actual accompanying Sox2 channel. Second, as a control in which the relationship between dendrite area and neighboring soma position was severed, we used “unmatched” shuffled images in which the dendritic ROI was placed over non-matching Sox2 images. These unmatched control images were generated by transposing the dendritic ROI onto the Sox2 channel from other fields of view from the same dataset – i.e., dendrite polygons from wild-type ON starbursts were transposed onto wild-type ON Sox2 images, while *Megf10* mutant OFF polygons were transposed onto mutant OFF Sox2 images. Typically, each arbor was placed onto the Sox2 array of all other images from the same dataset, although there were rare instances where a certain Sox2 array was not used. This procedure generated a family of unmatched control images for each dendritic arbor (*n* = 17–38 simulations per arbor, varying based on the number of real images in each dataset; see figure legends for precise numbers). For some analyses (Figure S3) we also generated “mutant on wild-type” unmatched controls in which *Megf10* mutant polygons were transposed onto wild-type Sox2 arrays. After generating the unmatched control images, the number of Sox2 cells fully enclosed by the arbor polygon was manually counted in each simulation, using the methodology noted above for the real images. We then calculated unmatched enclosure rates for each individual arbor by averaging across the family of simulations generated from that arbor.

The unmatched dataset was used to test whether the frequency of Sox2 soma enclosure observed in the real data is lower than the frequency expected by chance. Box and whisker plots comparing enclosure numbers for real and unmatched arbors (e.g., Figures 3C and 6H) were generated using the full simulation dataset. The individual arbor unmatched averages were used for two analyses: 1) frequency distribution histograms, which plotted the frequency with which *n* somata were enclosed by an individual arbor (e.g., Figures 3D, 3E, 6I, and 6J); and 2) calculation of the enclosed cell index for each arbor (e.g., Figures 3F and 6K). These two analyses are described below in further detail. For these two analyses we excluded unmatched images that did not enclose any Sox2 cells; this was necessary because real images always contained at least one Sox2 cell – i.e., the soma of the reference mGFP^+^ cell. Therefore, without excluding zero values, the unmatched datasets were systematically skewed toward smaller values. Moreover, without this exclusion, chi-squared tests comparing enclosed cell proportions from real and unmatched datasets (see below) would not be valid because the “0” bin would differ between these groups for artificial reasons. Notably, even with the skew toward smaller values introduced by inclusion of zeroes in the unmatched datasets, we still found more enclosed cells in the unmatched groups (Figures 3C and 6H).

##### Frequency distribution histograms.

The “real” curves in these histograms represent the fraction of arbors that enclosed exactly *n* Sox2^+^ somata within the real images. To obtain the “unmatched” curves, we began by calculating the frequency with which each individual dendritic arbor ROI enclosed *n* Sox2^+^ somata across all of the unmatched control images using that arbor. This table of per-arbor enclosure frequencies was then used to calculate the mean (±SEM) frequency of enclosing *n* somata across all arbors in a given dataset, which is the value plotted in the figures (e.g., Figures 3D and 3E). Chi-squared tests were used to test for differences between real and unmatched curves.

##### Enclosed cell index.

To compare real Sox2 cell enclosure rates to chance rates at the individual arbor level, we calculated the enclosed cell index for each mGFP^+^ arbor. This was defined as:

(mean enclosed cells from individual arbor unmatched images) – (real enclosed cell number)

This index will be equal to zero if the number of cells enclosed by a given arbor in its real and unmatched images is the same. Deviations from zero indicate more cells enclosed by either real or unmatched arbors (positive = more in unmatched; negative = more in real). To evaluate deviations from zero, the index was plotted for each cell, and the median value was compared to zero using a Wilcoxon one-sample test. This procedure is tantamount to performing a Wilcoxon matched-pairs test comparing the number of Sox2 cells enclosed by real arbors vs. their unmatched controls.

#### Analysis of dendro-somatic starburst contacts

For quantitative analysis of dendrite-soma contacts in the INL: Confocal en-face images of Chat:mGFP^+^ OFF starburst cells (P0-P1) were acquired from mice that also carried a single copy of the *Megf10*^*lacZ*^ allele, such that the full starburst array could be revealed using antibodies to β-galactosidase (βgal). Using Olympus FV300 or FV3000 confocal microscopes, Z-stacks were acquired that encompassed the full arbor of the GFP^+^ reference cell; the portion of the GFP^+^ arbor within the INL was then identified by referencing the βgal channel, which showed starburst dendrites at the IPL level and starburst somata at the INL level. The trajectory and termination site of each GFP^+^ arbor was examined in three dimensions, using 3D reconstructions and orthogonal views, as necessary, to score it as to whether it terminated upon a neighboring βgal^+^ cell. As a negative control measuring the frequency of soma contact that may be expected by chance, the same analysis was performed on Z-stacks wherein the GFP channel was flipped about the horizontal and vertical axes, as previously described.^4^ The fraction of GFP^+^ arbors contacting βgal^+^ somata was then calculated for both real and flipped conditions. This analysis was enabled by the relative sparseness and simplicity of the INL dendritic arbors. When we attempted a similar analysis for GFP^+^ IPL arbors, which were ramified in a much more elaborate manner, we were unable to accurately count and score all of the numerous and finely branched arbor tips.

For representative images of dendrite-soma contact: *En face* images are Z-projections of a small number of Z-planes encompassing the relevant part of the arbor – i.e., either the portion of the arbor ramifying within the IPL or the portion ramifying within the INL. These projections typically encompass 2–10 μm of Z distance. Cross-sections show Z-projections encompassing the arbors of specific cells chosen as representative examples – typically 10–15 μm of Z distance. For these illustrations of dendrite-soma contacts, we always confirmed in the full z stack that the touching dendrite and soma were in fact present within the same Z plane. This step was essential to exclude the possibility that Z projections might create a false impression of contact between starburst elements in different Z planes. Definitive confirmation of dendrite-soma contact was only possible when a GFP^+^ dendrite contacted a GFP-netive cell body. However, in Megf10:mGFP bulk labeled tissue, it was also possible to appreciate likely contacts between two GFP^+^ cells in cases where a single, fine, unfasciculated process connected two cell bodies. Such cases were annotated as putative dendrite-soma contacts. Putative and definitive contacts were marked with different colored arrows in Figure 2.

#### Analysis of soma positioning in Chat-Megf10-cKO mice

To identify individual MEGF10 mutant cells in Chat-Megf10-cKO mice, cross-sections from P3 retinas were stained with antibodies to GFP (marking Cre reporter-positive cells) and MEGF10. Two channel confocal Z-stacks were collected by confocal microscopy (Olympus FV300 or FV3000 with a UPlanS APO 60x/1.3 Sil objective). The distance of GFP^+^ cells (with or without MEGF10) to the IPL was measured in Fiji by drawing a line beginning at the edge of the soma proximal to the IPL, and ending at the IPL border. Cell distances were scored blind to MEGF10 expression status.

#### Histology and immunohistochemistry

##### Retinal cross sections:

Mice were anesthetized by hypothermia (P0 and P5 mice) or isoflurane (all other ages) and euthanized by decapitation. Eyes were enucleated, washed in PBS, and fixed in PBS containing 4% formaldehyde (pH 7.5) for 1.5 h at 4°C. After fixation, eyes were washed with PBS (2x) and stored in PBS containing 0.02% sodium azide at 4°C until further processing. The eyecup was isolated and sunk in PBS containing 30% sucrose, then embedded in Tissue Freezing Medium (VWR) before being frozen in 2-methylbutane chilled by dry ice. Tissue sections were cut on a cryostat to 20 μm and mounted on Superfrost Plus slides and dried on a slide warmer. For antibody labeling, slides were washed for 5 min with gentle agitation in PBS to remove embedding medium and blocked for 1 h in PBS +0.3% Triton X-100 (PBS-Tx) containing 3% normal donkey serum. Primary antibodies were diluted in blocking buffer, added to slides, then incubated overnight at 4°C. Slides were washed with PBS 3X for 10 min followed by incubation with secondary antibodies diluted in PBS-Tx for 1–2 h at RT. Slides were washed again with PBS 3X for 10 min then coverslipped using Fluoromount G.

##### Retinal whole-mounts:

After obtaining eyecups as described above, retinas were dissected free of the eyecup, washed in PBS, then blocked for 3 h with agitation at 4°C in blocking buffer (constituted as described above). Primary antibodies were diluted in blocking buffer, added to retinas, and incubated for between 5 and 7 days on a rocker at 4°C. After primary staining, retinas were washed 3 times with PBS over 2 h and then incubated in secondary antibodies (diluted in PBS-Tx). Secondary staining was performed overnight at 4°C with gentle agitation. Retinas were again washed in PBS (3x) over 2 h on a room temperature rocker. For mounting on slides, four 90° radial incisions were made, approximately 1/3 the radius of the retina. Retinas were then flattened on nitrocellulose paper (Millipore) photoreceptor side down and coverslipped with Fluoromount G (Southern Biotech).

### QUANTIFICATION AND STATISTICAL ANALYSIS

Density recovery profile calculations were performed using WinDRP software. Voronoi domain territory sizes were obtained using Fiji/ImageJ. All other statistical analyses were performed in JMP 12 (SAS Institute) or GraphPad Prism 10. Parameters of statistics (i.e., sample size, tests conducted and *p*-values) can be found within figures or figure legends. Post-hoc tests following ANOVA analysis were corrected for multiple comparisons. Alpha threshold for determining statistical significance was *p* ≤ 0.05 unless otherwise stated. *p*-values below the alpha threshold are denoted using red text in the figures; non-significant *p*-values are given in black text. For analysis of Sox2 cell enclosure by starburst dendritic arbors, nonparametric statistical tests (Mann-Whitney U test, Wilcoxon test) were used because of the non-Gaussian distribution of values (note skewed distributions in Figures 3D and 6I).

## Supplementary Material

1

## Figures and Tables

**KEY RESOURCES TABLE:** 

REAGENT or RESOURCE	SOURCE	IDENTIFIER

Antibodies		
rabbit anti-MEGF10	Millipore	RRID:AB_11204003; Cat# ABC10
rabbit anti-Sox2	Abcam	RRID: AB_2341193; Cat# ab97959
goat anti-Sox2	Santa Cruz	RRID:AB_2286684; Cat# sc-17320
rabbit anti-GFP	Millipore	RRID:AB_2630379; Cat# AB3080P
chicken anti-GFP	Life Technologies	RRID:AB_2534023; Cat# A10262
rabbit anti-β-galactosidase	gift of Joshua Sanes; Kay et al.^15^	N/A
goat anti-ChAT	Millipore	RRID: AB_2079751; Cat# AB144P
rat anti-RFP, clone 5F8 (detects tdTomato)	ChromoTek	RRID:AB_2336064
guinea pig anti-RBPMS	Millipore	RRID:AB_2687403; Cat# ABN1376
donkey anti-rabbit Alexa 488	Jackson Immunoresearch	RRID:AB_2313584; Cat# 711–545-152
donkey anti-goat Alexa 488	Jackson Immunoresearch	RRID:AB_2336933, Cat# 705–545-147
donkey anti-chicken Alexa 488	Jackson Immunoresearch	RRID:AB_2340375, Cat# 703–545-155
donkey anti-goat Cy3	Jackson Immunoresearch	RRID:AB_2307351; Cat# 705–165-147
donkey anti-rat Cy3	Jackson Immunoresearch	RRID:AB_2340667, Cat# 712–165-153
donkey anti-rabbit Cy3	Jackson Immunoresearch	RRID:AB_2307443, Cat# 711–165-152
donkey anti-guinea pig Cy3	Jackson Immunoresearch	RRID:AB_2340460; Cat# 706–165-148
donkey anti-goat Alexa 647	Jackson Immunoresearch	RRID:AB_2340437; Cat# 705–605-147
donkey anti-rabbit Alexa 647	Jackson Immunoresearch	RRID:AB_2492288, Cat# 711–605-152
donkey anti-guinea pig Alexa 647	Jackson Immunoresearch	RRID:AB_2340476; Cat# 706–605-148
donkey anti-mouse Alexa 647	Jackson Immunoresearch	RRID:AB_2340863; Cat# 715–605-151
donkey anti-rat Alexa 647	Jackson Immunoresearch	RRID:AB_2340694; Cat# 712–605-153
Chemicals, peptides, and recombinant proteins		
Normal donkey serum	Jackson Immunoresearch	RRID:AB_2337258; Cat# 017–000-121
16% Paraformaldehyde	Electron Microscopy Sciences	Cat# 15710
Fluoromount G	Southern Biotech	0100–01
Hoechst 33258	Invitrogen	H21491
Experimental models: Cell lines		
R1 embryonic stem cells	ATCC	RRID:CVCL_2167
Experimental models: Organisms/strains		
mouse: *Megf10^tm1b(KOMP)Jrs^* (*Megf10^lacZ^* or *Megf10^−^*)	Kay et al.^15^	MGI:6194030
mouse: *Megf10^tm1c(KOMP)Jrs^* (*Megf10^flox^*)	Ray et al.^4^	MGI:6194031
mouse: *Megf10^tm1.1(cre)Jnk^* (*Megf10^Cre^*)	this study	MGI:6740603
mouse: *Chat^tm2(cre)Lowl^*	Jackson labs	RRID:IMSR_JAX:006410
mouse: Tg(ACTFLPe)9205Dym/J	Jackson labs	RRID:IMSR_JAX:005703
mouse: Tg(Pax2-cre)1Akg/Mmnc (Pax2-Cre)	Gift of Joshua Weiner; Ohyama and Groves.^30^	RRID:MMRRC_010569-UNC
mouse: *Atoh7^tm1Gla^* (*Math5T^−^*)	Gift of Tom Glaser; Brown et al.^33^	RRID:MMRRC_042298-UCD
mouse: *Gt(ROSA)26^Sortm4(ACTB-tdTomato,-EGFP)Luo^*	Jackson labs	RRID:IMSR_JAX:007576
mouse: Gt(ROSA)26Sor^tm1(CAG-EGFP)Blh^	Gift of Brigid Hogan; Rawlins et al.^48^	MGI:3850169
mouse: *Gt(ROSA)26Sor^tm14(CAG-tdTomato)Hze^*	Jackson labs	RRID:IMSR_JAX:007914
mouse: C57Bl6/J	Jackson labs	RRID:IMSR_JAX:000664
Oligonucleotides		
*Megf10^Cre^* genotyping primer F1 caacagcaccagcagcaacagcac	IDT	N/A
*Megf10^Cre^* genotyping primer R1 (used with primer 1F; gives 368 bp band on wild-type allele and fails on mutant alleles) gct ttc tgg tac acg tcc aac aac tgc tta tta gag tat ttc	IDT	N/A
*Megf10^Cre^* genotyping primer R2 (used with primer 1F; gives 418 bp band on mutant alleles and fails on wild-type allele) cct ggc gat ccc tga aca tgt cca tc	IDT	N/A
*Megf10Cre* genotyping primer F2 (used with primer R1; tests for removal of *Neo*. Gives 185 bp band for *Cre* allele; fails on *CreNeo* and wild-type alleles). gga aca aaa act tat ttc tga aga aga tct gtg aaa g	IDT	N/A
Neomycin cassette genotyping Fwd tgc tcc tgc cga gaa agt atc cat cat ggc	IDT	N/A
Neomycin cassette genotyping Rev (tests for presence of *Neo*; 380 bp band with Fwd primer) cgc caa gct ctt cag caa tat cac ggg tag	IDT	N/A
Recombinant DNA		
plasmid: pL253	Liu et al^49^	N/A
plasmid: pL253.02 *Megf10* knock-in targeting construct	this study	N/A
Software and algorithms		
Fiji/ImageJ	Schindlein et al.^50^	RRID:SCR_002285
WinDRP	Rockhill et al.^8^	N/A
Prism 10	GraphPad	N/A
JMP 12	SAS Institute	N/A
MATLAB 2018	MathWorks	N/A

## Highlights

- Regular spacing of the starburst amacrine cell mosaic is not established by dendrite tiling
- Starburst dendrites exclude neighboring somata to establish mosaic spacing
- Repulsion between starburst dendrites and somata is mediated by transcellular MEGF10 signals
- Loss of dendrite-soma exclusion in *Megf10* mutants abolishes starburst mosaic spacing
