# Supplementary material for: Retinal neurons establish mosaic patterning by excluding homotypic somata from their dendritic territories
Source: Cell Rep. Author manuscript; Available in PMC 2024 Sep 30. (PMC11440617; doi:10.1016/j.celrep.2024.114615)
Supplement: 1 [file NIHMS2019548-supplement-1.pdf]

Cell Reports, Volume *43*

Supplemental information

Retinal neurons establish mosaic patterning by excluding homotypic somata from their dendritic territories

Christopher Kozlowski, Sarah E. Hadyniak, and Jeremy N. Kay

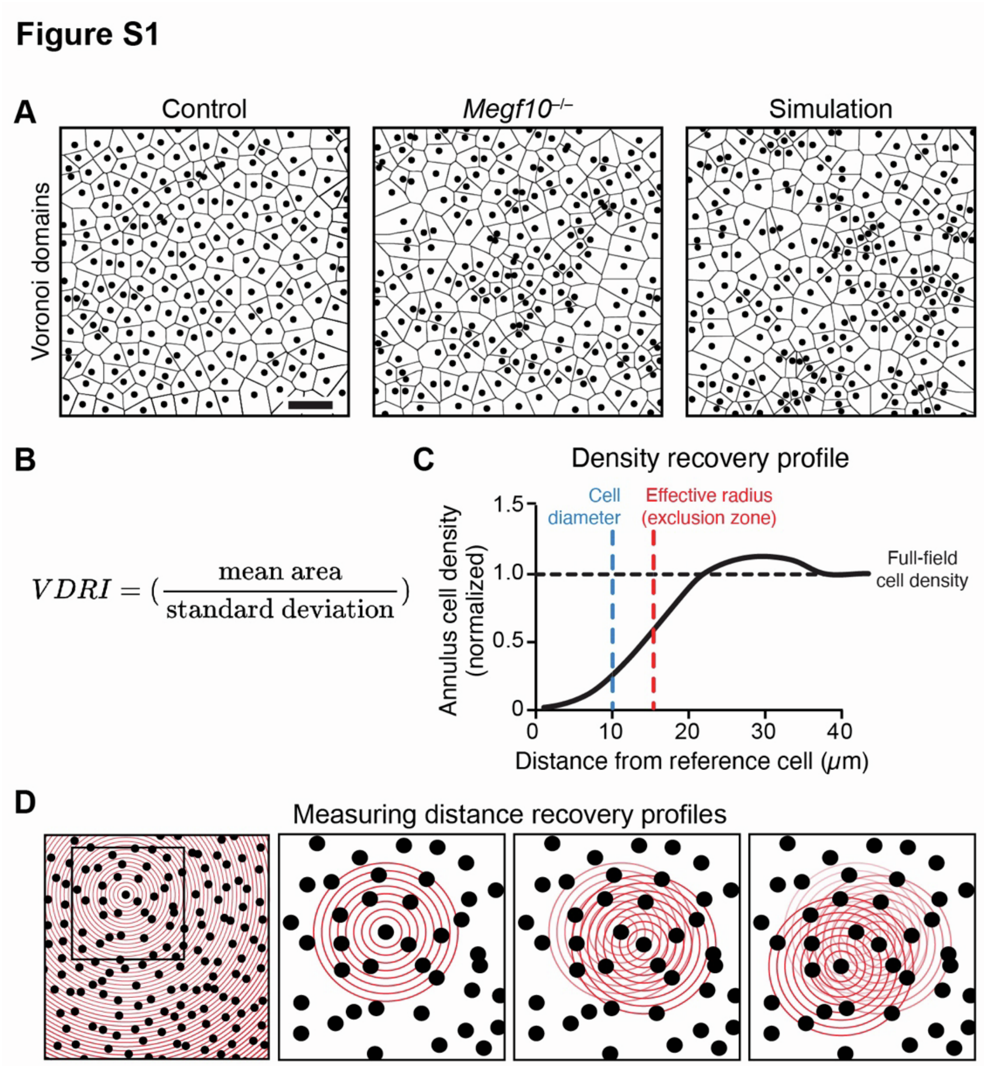

Figure S1 (related to Figures 1, 6 and 7): Methods used for analysis of starburst spatial patterning.

(**A,B**) Quantification of mosaic regularity using Voronoi domain regularity analysis. (**A**) Representative examples of OFF starburst soma locations (circles) and their Voronoi domains from P5 wild-type retina (left); P5 *Megf10* mutant retina (center); or a computer-generated random simulation (right). Circles represent cell locations and are sized to approximate the average diameter of P5 OFF starburst somata. Voronoi domains, defined as the set of points closest to any given cell, are indicated by lines/polygons. For left and center panels, cell locations were derived from real images like those in Fig. 1B. Random simulations (right) were generated using a Poisson point process, matching the cell size and cell density of P5 starbursts as measured from real images (see Methods). In wild-type arrays, the areas of the Voronoi domains are fairly uniform, reflecting the uniform distribution of starburst neurons in wild-type retina. By contrast, in *Megf10* mutants and in random simulations, Voronoi areas are more variable in size. (**B**) Formula for computing Voronoi domain regularity index (VDRI): The mean Voronoi area for a given image is computed and divided by the standard deviation of the area sizes. Note that Voronoi domains touching the edge of the field of view are excluded from the analysis.

(C,D) Quantification of exclusion zone sizes using the density recovery profile (DRP). (**C**) Schematic showing key features of the DRP plot. The plot shows how the density of cells within annuli of *d* distance from any given cell compares to the overall cell density within an image (horizontal dashed line). (**D**) Illustration of the procedure used to compute the DRP. For each cell in the array, a series of rings of increasing size (step size used here, 3-5 μm) are drawn with the index cell at the center. The density of cells lying within each annulus is computed. After the procedure is repeated for each cell in the array, the average cell density for each annulus is calculated, normalized to the overall cell density in the entire image, and plotted as in C.

In the example graph (C), which is a schematic illustrating typical starburst measurements, rings close to the reference cell have lower cell density than the overall image, indicating short-range cell-cell repulsion that prevents homotypic neighbors from settling near each other. At longer distances, annulus cell density approximates the overall cell density. The effective radius (i.e. exclusion zone) is measured as the midway point of the rising portion of the curve (red vertical dashed line). If cells are randomly positioned – i.e. they do not show local cell-cell repulsion or attraction – then the only constraint on their position is that two cells cannot occupy the same physical location in the 2D plane. As such, the exclusion zone size measured by DRP will be approximately equal to the average cell diameter (blue line). In our analysis of OFF starbursts, both real *Megf10* mutant arrays (A, center panel) and random simulated starburst arrays (A, right panel) had measured exclusion zones similar to the average starburst cell diameter (see Fig. 1F). For cell types that form mosaics, such as wild-type starbursts, exclusion zones are larger than the cell diameter (compare vertical dashed lines), which indicates presence of *bona fide* cell-cell avoidance.

The DRP can also be used to measure cell-cell attraction, which is indicated by the presence of annuli with a higher cell density than the overall image (i.e. above the horizontal dashed line, not shown here but see Fig. 7D). In these cases, cells may be pulled out of their 2D plane into a clump, which can lead to exclusion zone size measurements that are lower than the average cell diameter – as cells will appear to overlap in the photomicrographs used for DRP analysis. This phenomenon was observed for ON starbursts in *Megf10* mutants: As shown in Fig. 6D, the exclusion zone size was lower for real ON mutant cells than for simulations.

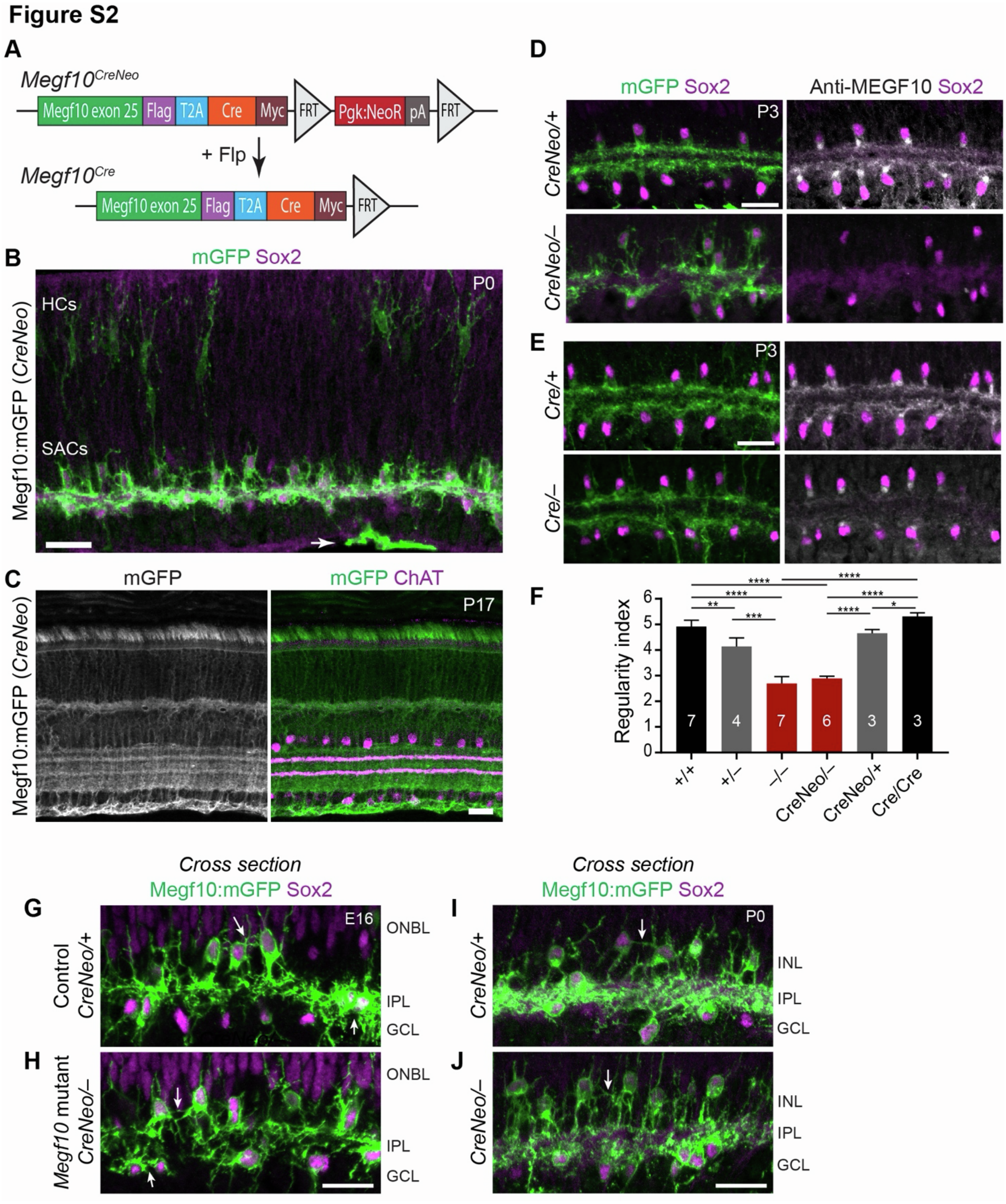

Figure S2 (related to Figure 2): Generation of *Megf10*^{CreNeo} and *Megf10*^{Cre} mice.

(**A**) Schematic of *Megf10* knock-in alleles. *Megf10*^{CreNeo} knock-in mice were produced by inserting a Flag-T2A-Cre-Neomycin (Neo) cassette in place of the endogenous *Megf10* stop codon within Exon 25. To generate the *Megf10*^{Cre} allele, *Megf10*^{CreNeo} mice were bred to a FLP-

recombinase germline deleter mouse, thereby removing the Neo sequence via Flp-FRT recombination.

(**B,C**) *Megf10*^{CreNeo} expression pattern was assessed by crossing to a mGFP Cre reporter line. At P0 (B), most Sox2^{+} starburst amacrine cells (SACs) are GFP^{+}, as are horizontal cells (HCs) in outer retina. Occasional GFP-negative Sox2 cells are observed. Sparse labeling is also seen in nerve fiber layer astrocytes (arrow). At P17 (C), Müller glia labeling has become prominent, although ChAT^{+} starburst cells remain GFP^{+}. Expression by these other cell types does not compromise our ability to visualize starburst morphology during the E16-P3 period because they are either located in other retinal layers (horizontal cells and astrocytes; B) or because they only express *Megf10* later in development (Müller cells, C).

The Cre expression pattern (B,C; main Fig. 2K,L) is entirely consistent with our previous studies of endogenous *Megf10* expression: We showed that *Megf10* RNA and/or protein is expressed by starburst cells and nerve fiber layer astrocyte precursors starting at E16; by horizontal cells starting at P0; and by Müller glia starting around P7[4,15]. Thus, Cre expression faithfully reports the endogenous *Megf10* pattern.

(**D,E**) Comparison of *Megf10*^{CreNeo} (D) and *Megf10*^{Cre} (E) alleles at P3. Both alleles drive expression of mGFP reporter in most Sox2^{+} starburst cells. The *CreNeo* allele (D) does not support expression of MEGF10 protein, as *CreNeo/–* mice lack MEGF10 immunoreactivity and show IPL arborization phenotypes typical of *Megf10*$^{–/–}$ animals[4]. *Cre/–* mice have normal starburst anatomy and still express MEGF10 protein (E). Note that soma-layer arbors (Fig. 2G-I) are absent by P3.

(**F**) Mosaic regularity of P17 OFF starburst cell arrays, measured by Voronoi domain regularity index, in mice carrying various *Megf10* alleles. The *CreNeo* allele behaves similarly to the null (–) allele, causing mosaic defects, whereas the *Cre* allele behaves similarly to the wild-type (+) allele. Statistics: One-way ANOVA with Tukey's post-hoc test. Colors indicate pairs of genotypes that are not significantly different from each other (+/+ vs. *Cre/Cre*, $p = 0.17$; +/– vs. *CreNeo/+*, $p = 0.58$; –/– vs. *CreNeo/CreNeo*, $p = 0.74$) , but which are significantly different from the other color groups. *$p = 0.018$; **$p = 0.0003$; ***$p < 1 \times 10^{-6}$; ****$p < 1 \times 10^{-7}$. Sample sizes: n = 6 animals (+/+; CreNeo/–) or n = 4 (others).

(**G-J**) Anatomy of starburst dendrites at E16 and P0 in control and *Megf10* mutant mice, assessed using Megf10:mGFP bulk labeling. At E16 (G,J), starburst neurons interconnect their dendrites both within soma layers (arrows) and IPL. Anatomy is similar in both genotypes. At P0 (H,I), IPL innervation has increased substantially in controls as compared to E16. By contrast, *Megf10* mutants show a reduced density of dendrite labeling in IPL, consistent with our prior findings using different starburst labeling strategies[4]. Soma layer arbors are still evident in both genotypes. Note that the P0 wild-type image from Fig. 2G is reproduced here (I), to enable comparisons with P0 mutant.

Error bars, SD. Scale bars = 25 μm.

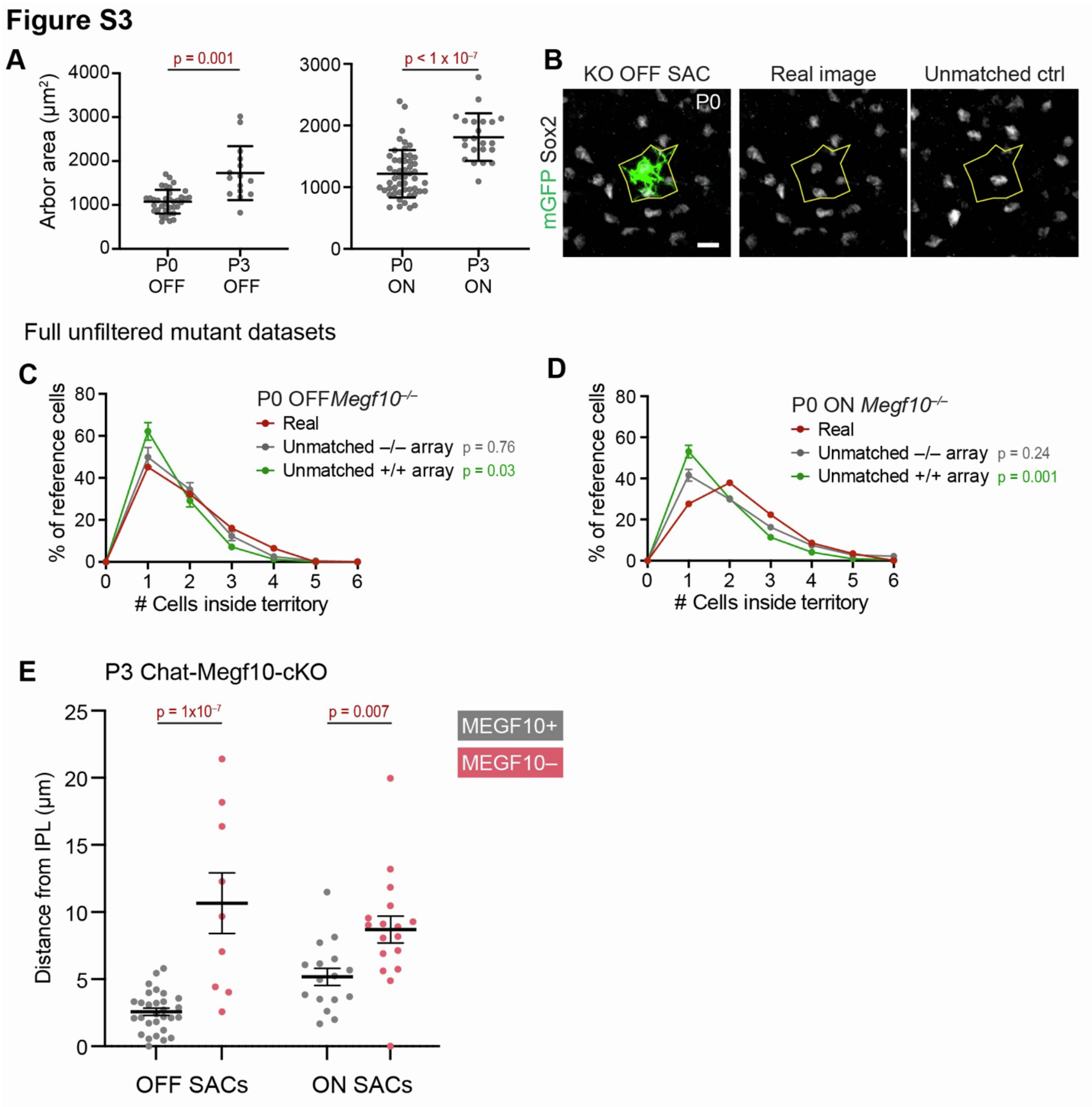

Figure S3 (related to Figures 3, 4, and 6): Additional analysis of starburst dendritic arbor size and soma exclusion.

(**A**) Wild-type starburst arbors are larger on average at P3 than at P0. Left, OFF starbursts; right, ON starbursts. Statistics: two tailed t-test, p-values shown on graph. Sample sizes (OFF): P0, n = 36; P3, n = 15. Sample sizes (ON): P0, n = 52; P3, n = 22. Error bars, mean ± SD.

(**B**) Example image showing a *Megf10*$^{-/-}$ OFF starburst with a small dendritic territory; this cell was outside the 1-standard-deviation cutoff used for the analysis in Figure 4. Left, center panels show real image; right panel shows unmatched control image in which the dendritic polygon was placed onto a mutant OFF starburst array from a different retinal location. Sox2 marks neighboring starburst somata. Scale bar, 10 µm.

(**C,D**) Frequency distribution histograms showing $Sox2^+$ cell enclosure for the full unfiltered *Megf10*$^{-/-}$ OFF (C) or ON (D) datasets. To measure enclosure frequencies expected by chance, we generated two types of unmatched controls: one in which real mutant arbor polygons were placed onto mutant (–/–) Sox2 arrays (gray; this was the methodology used for Fig. 3M and Fig. 6P), and one in which these polygons were placed onto wild-type (+/+) Sox2 arrays (green). Real and mutant-unmatched curves were not statistically distinguishable, consistent with a lack of dendrite-soma exclusion in mutants. This finding is in accord with our results using size-matched filtered datasets (Figs. 3M and 6P). If the +/+ unmatched curve is taken as the chance enclosure rate, it would appear that more cells are enclosed by real mutant arbors than expected by chance. This result may suggest possible starburst aggregation in mutants, or it may mean that using the mutant Sox2 array provides a more accurate measure of the chance enclosure rate. However, it is not consistent with presence of dendrite-soma exclusion, which would have caused the real mutant curve to be shifted left relative to the control distribution. Thus, regardless of whether the filtered or full datasets are used, and regardless of whether mutant unmatched simulations are generated using mutant or wild-type Sox2 arrays, our conclusion that dendrite-soma exclusion is absent in mutants remains unchanged.

Sample sizes: mutant OFF n = 29 real arbors, 20 unmatched images per arbor; mutant ON n = 58, 32 unmatched images per arbor. Since “zero enclosed cells” is an impossible value for the real data (due to the presence of the reference cell body), we excluded zeroes counted from unmatched simulations (final sample sizes: OFF –/– unmatched, n = 479; OFF +/+ unmatched, n = 777; ON –/– unmatched, n = 1,449; ON +/+ unmatched, n = 1,786). Statistics, chi-squared tests comparing the real distribution to the specified unmatched control distribution. P-values shown on graph. Error bars, mean ± SEM.

(**E**) ON starburst soma positioning phenotype in Chat-Megf10-cKO mice. Graph shows distance from IPL for individual Chat:mGFP cells in cKO retinas that were scored for presence of MEGF10 protein as in Fig. 4D. OFF data is replotted from the main figure (Fig. 4G) to facilitate comparison with ON data. Whereas $MEGF10^+$ cells are located in a single stratum of the INL or GCL close to the IPL, cells lacking MEGF10 tend to be excluded from this 2D plane and instead occupy more distant positions. Sample sizes/statistics: OFF cells as in Fig. 4G; ON cells, $MEGF10^+$ n = 16; $MEGF10^-$ n = 17; two-tailed T-test, P-value shown on graph. Error bars, mean ± SEM.
